# Temporal transcriptome profiling of floating apical out chicken enteroids suggest stability and reproducibility

**DOI:** 10.1186/s13567-023-01144-2

**Published:** 2023-02-15

**Authors:** Tessa J. Nash, Katrina M. Morris, Neil A. Mabbott, Lonneke Vervelde

**Affiliations:** Division of Immunology, The Roslin Institute, R(D)SVS, University of Edinburgh, Midlothian Edinburgh, UK

**Keywords:** Chicken, 3D organoid, apical-out, transcriptome, intestine, stem cell

## Abstract

Enteroids are miniature self-organising three-dimensional (3D) tissue cultures which replicate much of the complexity of the intestinal epithelium. We recently developed an apical-out leukocyte-containing chicken enteroid model providing a novel physiologically relevant in vitro tool to explore host–pathogen interactions in the avian gut. However, the replicate consistency and culture stability have not yet been fully explored at the transcript level. In addition, causes for the inability to passage apical-out enteroids were not determined. Here we report the transcriptional profiling of chicken embryonic intestinal villi and chicken enteroid cultures using bulk RNA-seq. Comparison of the transcriptomes of biological and technical replicate enteroid cultures confirmed their high level of reproducibility. Detailed analysis of cell subpopulation and function markers revealed that the mature enteroids differentiate from late embryonic intestinal villi to recapitulate many digestive, immune and gut-barrier functions present in the avian intestine. These transcriptomic results demonstrate that the chicken enteroid cultures are highly reproducible, and within the first week of culture they morphologically mature to appear similar to the in vivo intestine, therefore representing a physiologically-relevant in vitro model of the chicken intestine.

**Supplementary Information:**

The online version contains supplementary material available at 10.1186/s13567-023-01144-2.

## Introduction

Organoids bridge the gap between traditional cell-lines and in vivo studies and facilitate research into multicellular biological mechanisms. The transcriptional analysis of enteroids has provided a greater understanding of the maturation stage, functions and responses of these in vitro culture models, as well as providing opportunities to study rare cell types [1]. Several RNA-seq datasets describing mammalian gel-embedded enteroids have already been published [2–4], but detailed analysis of the transcriptomes of avian enteroid cultures is lacking. Most RNA-seq studies of the chicken intestine have focused on functionality-related genes involved in digestion and absorption of energy and nutrients [5–7] and host–pathogen responses [8, 9] in mature birds. However, the late-embryonic to post-hatch gut undergoes immense changes in growth, metabolism and development. Analysis of the transcriptional changes in these critical life-stage processes could help identify novel methods to improve nutrition and gut-barrier function in the post-hatch chick [10–12].

Typically, gel-embedded enteroid crypts feed into a central functional lumen and are lined by a single layer of highly polarised epithelial cells. The apical brush borders of the epithelial cells face the lumen, with the basolateral surfaces in contact with the extracellular matrix scaffold [3]. This “basal-out” orientation means the luminal surface, which interacts with nutrients, toxins and microorganisms, is not directly accessible. Co et al. [13] demonstrated that by removing the extracellular matrix they could reverse the epithelial polarity of enteroids, allowing easy access to the apical surface. Their “apical-out” method involved an initial embedding step where the human and murine enteroids were established within Matrigel (a solubilized extracellular matrix secreted by Engelbreth-Holm-Swarm mouse sarcoma cells) for 7–20 days before moving them into a suspension. The first step appears necessary for mammalian enteroids to successfully retain their structural integrity in suspension. The mammalian apical-out enteroids were not described as surviving past 5 days in suspension culture and the ability to passage enteroids in this orientation does not appear possible. This technique has also been repeated in porcine enteroids where, similar to the human and mouse cultures, ~20% of enteroids did not reverse their polarisation [14]. This raises concerns with reproducibility of these suspension cultures and, alongside the loss of enteroid viability after a few days, restricts longer term use of this technique (reviewed in [15]).

We recently published a protocol to culture floating apical-out avian enteroids directly from embryonic intestinal villi tips and showed they reflect the cellular diversity and barrier function of the chicken intestinal epithelium [16]. The epithelium of the tips seals over in culture, enclosing an inner core of lamina propria, which likely supplies many of the stem cell niche factors. This inner core also contributes to the unique presence of functional intra-epithelial and lamina propria leukocytes. These avian enteroids develop more rapidly than their mammalian counterparts and appear to remain viable for up to 2 weeks in culture. We have also developed enteroids from mature birds however, as they have reduced longevity and budding compared to those developed from embryonic tissue, we have not explored these cultures in detail [16].

To demonstrate that apical-out chicken enteroids are a robust and reproducible cell culture model it is necessary to provide evidence of transcriptional homogeneity between biological and experimental replicates. Use of starting material (embryos) from the same breed and age and consistent culture conditions helps to reduce biological variation amongst enteroid cultures. However, it is uncertain whether inherent (epi) genetic and environmental variation among individual embryos renders individual biological replicates highly heterogeneous. Since these enteroid cultures could represent an important in vitro system for a diverse array of nutritional, pharmaceutical and host–pathogen studies, it is also necessary to have a detailed understanding of their cellular and transcriptional similarity to the avian intestine in vivo. Just like apical-out mammalian enteroid cultures, and despite many passage technique and supplementary growth factor trials, the floating chicken enteroids were unable to be indefinitely propagated. A detailed chronological study of the enteroid transcriptome could also identify the molecular mechanisms that limit their long-term culture.

In the current study, using floating apical-out avian enteroids derived from embryonic day 18 (ED18) chicken intestinal villi, we provide an in-depth analysis of their transcriptional responses over a 7 day culture period. The initial aim was to assess for homogeneity between replicates in order to demonstrate the highly reproducible consistency of this culture method. These data were also used to determine the period of stable gene expression in the cultures and identify the optimal time-window for experimental studies. We also aimed to describe the functional maturation of the enteroids compared to the in vivo intestine post-hatch at the transcript level. Our final aim was to identify putative transcriptional changes in floating apical-out enteroids that may underlie their inability to be indefinitely propagated. The results suggest that the chicken enteroid cultures are transcriptionally highly reproducible, and within the first week of culture the enteroids morphologically mature similar to the chick’s intestine. Therefore the apical-out chicken enteroids represent a physiologically-relevant in vitro model of the chicken intestine.

## Materials and methods

### Animals

Enteroid experiments were performed using ED18 Hy-Line Brown chickens (*Gallus gallus*; 3 days before hatching) obtained from the National Avian Research Facility, Edinburgh, UK. In addition, 8 week old Hy-Line Brown chickens were used for comparative studies. This breed and age of adult chicken were used in line with the 3Rs as they were concurrently in use by other groups. Ethical approvals were obtained from The Roslin Institute’s and University of Edinburgh’s Animal Welfare Ethics Review Board. All animals were housed in premises licensed under a UK Home Office Establishment License in full compliance with the requirements of the Animals (Scientific Procedures) Act 1986 and with approval from The Roslin Institute Animal Welfare Ethics Review Board.

### Villus isolation and enteroid culturing

Isolation of avian ED18 intestinal villi containing stem cells and generation of floating chicken enteroids was performed as described previously [16]. In brief, the small intestine (from duodenum to ileocaecal junction) was removed post-mortem, cut into 5 mm sections and washed in Ca^2+^- and Mg^2+^-free Phosphate-Buffered Saline (PBS). The tissue was digested in Dulbecco’s Modified Eagle’s Medium (DMEM) (Thermo Fisher Scientific (TFS)) with 0.2 mg/mL Collagenase from *Clostridium histolyticum* Type IA (Merck) at 37 °C for 50 min. The tube was shaken vigorously, supernatant collected and these steps were repeated to generate 4 fractions. Fractions were centrifuged at 100 *g* for 4 min and crypts/villi were plated out in Advanced DMEM/F12 (TFS) supplemented with 10 mM HEPES (TFS), 2 mM L-Glutamine (TFS), 50 U/mL Penicillin/Streptomycin (Merck) and 2% B27 supplement (50X; TFS). Culture of avian enteroids occurred at 37 °C, 5% CO_2._

### RNA-seq experimental design

A total of 3 biological replicates (each culture composed of 3 × ED18 Hy-Line chickens) were used for the RNA-seq analysis. This was needed to allow enough ED18 enteroids to be available for sampling for each technical replicate at each time point. The isolated villi from each biological replicate were seeded (at 100 villi/well) in two identical 24-well tissue culture plates which were cultured in identical conditions. Each culture plate was treated as a technical replicate, therefore there were 2 technical replicates for each biological replicate. For sample collection, 8 wells per plate were collected at each of the defined time points (freshly isolated villi defined as 0 h, 1 day, 3 day, 4 day and 7 days of culture) and pooled to form one sample for transcriptomic analysis.

### RNA sequencing and analysis

Enteroid total RNA was extracted and the quality and concentration assessed. Technical validation of the samples by tape-station confirmed they had good RNA integrity (RIN 6.1–9.6) and concentration (104–427 ng/µL) (Additional file 1). The libraries were sequenced, reads trimmed and aligned as previously described [16]. In brief, obtained reads were trimmed for quality and to remove adaptor sequences using Cutadapt. Reads after trimming were required to have a minimum length of 50 bases. Paired-end reads from Illumina sequencing were aligned to the *Gallus gallus* genome (Gallus_gallus-5.0) using STAR. The annotation used for counting was the standard GTF-format annotation for that reference (annotation v91). Raw counts for each annotated gene were obtained using the featureCounts software (v1.5.2).

The Principal Component Analysis (PCA) was constructed using normalised and filtered expression data from the unmerged technical replicates. *P*-values were obtained from an analysis of variance (ANOVA) test which assessed the associations between continuous value ranges in principal components and categorical experimental variables (group, pool and time). The differential gene expression analysis was performed as previously described [16], in brief differential gene expression analysis was performed within the Bioconductor edgeR package (v3.16.565). Comparison of the embryonic enteroid transcriptome at 0–7 days post cultivation revealed that there were no differentially expressed genes between the technical replicates (FDR < 0.05), demonstrating the consistency and reproducibility of the enteroid system. The sumTechReps function in EdgeR was used to merge technical replicates. The raw counts table was filtered to remove genes consisting predominantly of near-zero counts, filtering on counts per million (CPM) to avoid artefacts due to library depth. Statistical assessment of differential expression was carried out with the likelihood-ratio test. Differentially expressed genes were defined as those with FDR < 0.05 and logFC > 2. Heatmaps were constructed in R using the pheatmap package (v1.0.1066).

The annotated freshly isolated villi (0 h) and enteroid RNA seq normalised data sets were imported into the bioinformatics tool Graphia (Kajeka, Edinburgh, UK) [17–19] and a pairwise transcript-to-transcript Pearson’s correlation matrix was calculated as previously described [2]. The technical replicates were merged (average rather than sum due to the absence of the COB_2_168 hr sample) for this part of the analysis. A Pearson correlation coefficient cut-off threshold of *r* ≥ 0.95 was selected. The resulting graph was clustered using the Markov Clustering algorithm at an inflation value (which determines cluster granularity) of 2.2. The first 50 clusters (by number of genes) were manually grouped into similar gene expression profiles in relation to time points of culture, and each cluster location was identified on the network graph.

To enable functional annotation and interpretation of the clusters, the top 20 significantly overrepresented gene ontology (GO) within each expression profile group were identified using the Molecular Signatures Database [20]. To be accepted as significant, an over-represented GO terms needed a hypergeometric *P* < 0.05, and FDR < 0.05. Where the same genes within clusters represented several GO terms, only the most significant were selected for further consideration. Individual genes associated within the RNA-seq normalised dataset were also assessed for cellular functions and activities using a combination of literature review, Kyoto Encyclopaedia of Genes and Genomes (KEGG) Pathway database and GeneCards database. Heatmaps were constructed in R using the pheatmap package (v. 1.0.10) [21].

### RT-qPCR

Enteroid total RNA was extracted and the quality and concentration assessed. First-strand cDNA synthesis was performed using a SuperScript IV reverse transcription kit (Invitrogen) according to the manufacturer’s instructions. Primer design for qPCR and qPCR was carried as described in Borowska et al. [22]. Primers are described in Additional file 2.

### Transmission electron microscopy

Enteroids and villi were fixed in 3% glutaraldehyde in 0.1 M sodium cacodylate buffer, pH 7.3, for 2 h and processed as described previously [16]. Ultrathin sections(60 nm) were stained in uranyl acetate and lead citrate and imaged using a JEOL JEM-1400 Plus TEM and analysed using ImageJ (v1.52e, Fiji).

### Whole mount and immunohistochemical staining

Details of the sources, clone numbers and concentrations of the primary and secondary antibodies used for immunohistochemistry are provided in Additional file 3. Enteroids were fixed, blocked, stained and mounted as previously described [16]. Samples were visualised using a LSM710 or LSM880 Confocal Microscope (Zeiss) and processed using ImageJ v1.52e.

Proliferation was assessed in a 5-ethynyl-2′-deoxyuridine (EdU) incorporation assay as previously described [16].

### Statistics and reproducibility

The figure legends provide most details of sample sizes, replicates, number of repeats and statistics. Unless otherwise stated, experiment data is representative of at least 3 independent cultures each containing 2–3 embryos. Four pooled wells with ~100 enteroids/well were used for each histology sample. All measurements were recorded from distinct samples.

In the RNA-seq data each RNA sample was comprised of 8 pooled wells containing ~100 enteroids/well. An ANOVA test was performed to compare the associations between continuous value ranges in principal components and categorical experimental factors. Statistical assessment of the RNA-seq differential expression data was carried out using a quasi-likelihood F-test which accounts for uncertainty in estimating the dispersion for each gene. A Pearson’s correlation coefficient was used to measure the strength and direction of the linear relationship between genes/transcripts in the RNA-seq network analysis.

### Data records

The mRNA expression datasets for this study have been deposited in the European Nucleotide Archive (ENA) at EMBL-EBI under accession number PRJEB37491 and PRJEB51227.

## Results

### Transcriptomic analysis suggests enteroid cultures are highly reproducible

**Figure 1:**
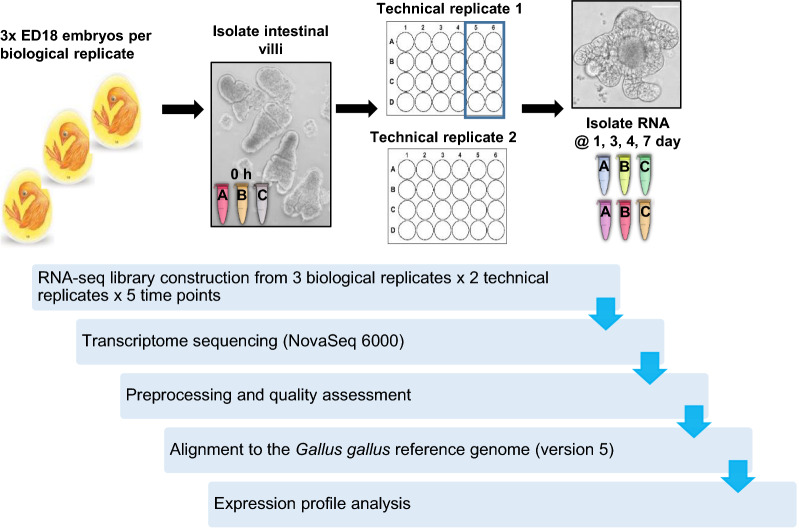
**Overview of experimental design and RNA-seq data analysis pipeline**. RNA was isolated from enteroids at 1, 3, 4 and 7 days and the freshly isolated ED18 intestinal villi (0 h). The validated RNA was sequenced on a Illumina NovaSeq 6000 system. All RNA-seq reads were preprocessed for a quality assessment. The filtered transcriptome reads were aligned to the genome and the expression profile was analysed.

Enteroid cultures were prepared from isolated villi of an individual embryo as described in [16], with three independent cultures pooled to make one biological replicate, and each culture split into two technical replicates after villus isolation (Figure 1). Samples for transcriptome analysis were collected at 0 h (freshly isolated ED18 intestinal villi) then at 1, 3, 4 and 7 days of culture. The 1 day time point was selected as it represents a time of initial rapid and major morphological change in the enteroids, days 3 and 4 were chosen as the cultures appear more morphologically stable at this time, and 7 day enteroids were of interest as this time point is typically used for passage of basal-out enteroids.

**Figure 2:**
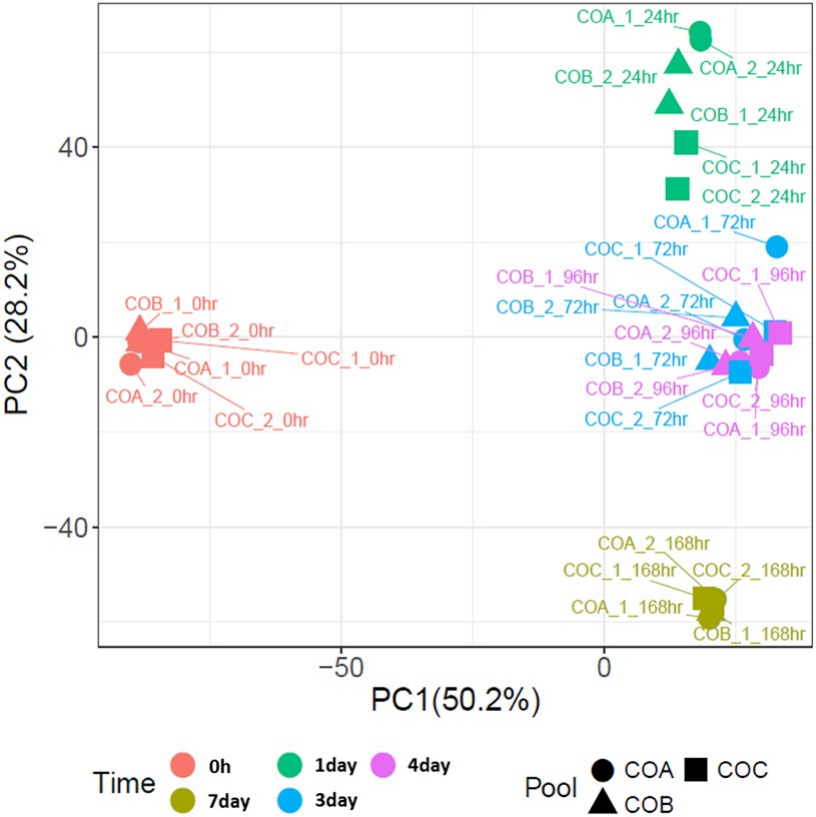
**Principle Component Analysis (PCA) of RNA-seq expression data**. Plot of the first and second principal components (PCs) from PCA using all samples. In PC1 and PC2, technical replicate per time point and time were the significant drivers for differences. Sample “COA_1_0hr” describes “Chicken Organoid, biological replicate A, technical replicate 1, 0 h time point” etc. The % values after the PCs indicate their contribution to the variability in the data.

To assess the reproducibility of the cultures, the degree of heterogeneity in the biological and technical replicate global transcriptional profiles was compared using a PCA. This showed that the biological and technical replicates tightly clustered by time point (Figure 2), confirming that the global transcriptomes of the enteroid culture replicates were highly consistent. The PC1 and 2 plot, where technical replicate per time point and time were the main drivers for the differences seen among the samples, showed clustering of the biological and technical replicates for each time point to independent locations on the plot, except for the 3 and 4 day replicates which occupied the same area, suggesting they shared a similar global transcriptional profile. However, defined clustering of ED18 intestinal villi (0 h) on the left side and the enteroid time points (1–7 day) on the right side of the PC1 axis is apparent which may represent the initial change from in vivo growth to in vitro culture.

### Early enteroid cultures continue the rapid development as seen in the chick

**Figure 3:**
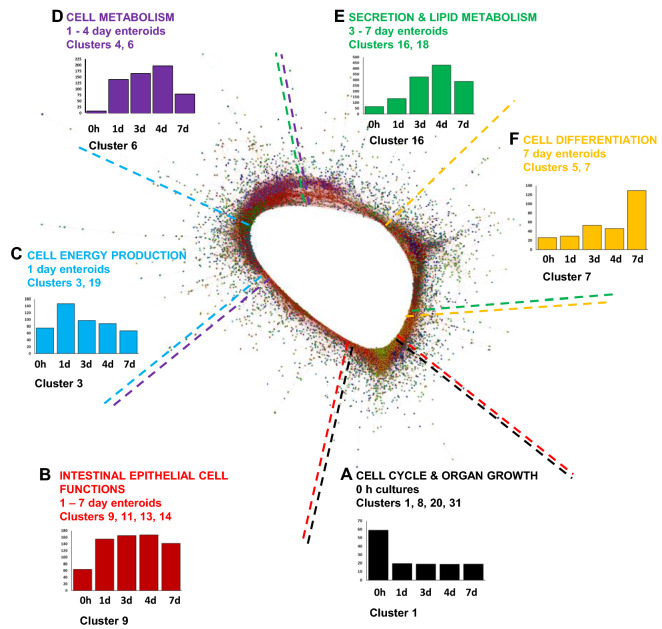
**Network analysis of ED18 intestinal villi-enteroid RNA-seq expression data**. Main component of the network graph derived from all five data sets samples. Here, the nodes represent transcripts (genes) and the edges represent correlations between individual expression profiles above r ≥ 0.95. Clusters that contain genes which are predominantly expressed at similar levels in all enteroids occupy different regions in the network graph. Coloured broken lines delineate these regions containing clusters of genes with similar mean expression profiles (**A**–**F**). The graph x axis shows the samples ordered from time of cultures. The *y* axis shows the mean expression intensity (transcripts/million reads, TPM) for the cluster.

A gene-to-gene correlation network graph was created next using the bioinformatics tool Graphia in order to explore the functional maturation of the enteroids during culture. A gene-to-gene correlation matrix was calculated for each transcript across each of the mRNA-seq data sets using a Pearson correlation threshold of r ≥ 0.95. The resulting network graph is presented in Figure 3 and contains groups of genes (clusters) with similar co-expression profiles across all data sets at r ≥ 0.95. The genes within the top 50 clusters (containing ≥ 17 genes) are presented in Additional file 4. Clusters with similar mean expression profiles typically occupied similar regions of the network graph. The clusters predominantly expressed at 0 h (Figure 3A) are associated with cell cycle and organ growth, whereas clusters predominantly expressed at higher levels in 1–7 day enteroids (Figure 3B) are associated with intestinal epithelial-cell specific functions. The clusters associated with cell energy production, cell metabolism are predominantly expressed at 1–4 days in culture, and clusters predominantly expressed at 3–7 days in culture cell are associated with secretion and lipid metabolism were found from 1 to 7 days in culture (Figures 3C–E). Clusters predominantly expressed in 7 day enteroids (Figure 3F) are mainly associated with cell differentiation.

The mean gene expression profiles and representative human GO term enrichment annotations for the largest 50 clusters from the network graph are provided in Additional files 5 and 6. A summary of GO term enrichment annotations from select clusters was created for ease of reference which highlight processes and functions for cell cycle, tissue development and cell functions within the enteroid culture time points (Tables 1 and 2).

**Table 1:** **GO term enrichment annotations for network clusters grouped into similar time-point expression profiles.**

Cluster	GO term no	Significantly-enriched GO term (*p* < 0.05)	*p* value	Genes in gene set	Genes in cluster	Genes in overlap	% overlap
High villi (0 day)							
1	7049	CELL_CYCLE	4.30E-63	1864	1190	175	9.388412
1	51301	CELL_DIVISION	8.78E-36	598	1190	76	12.70903
1	9893	POSITIVE_REGULATION_OF_MULTICELLULAR_ORGANISMAL_PROCESS	1.71E-29	1825	1190	123	6.739726
1	15630	MICROTUBULE_CYTOSKELETON	6.93E-32	1220	1190	102	8.360656
1	22008	NEUROGENESIS	2.92E-34	1625	1190	123	7.569231
1	51276	CHROMOSOME_ORGANIZATION	3.53E-35	1223	1190	107	8.748978
1	45595	REGULATION_OF_CELL_DIFFERENTIATION	1.52E-30	1881	1190	127	6.751728
8	35295	TUBE_DEVELOPMENT	9.42E-10	1122	194	18	1.604278
8	2520	IMMUNE_SYSTEM_DEVELOPMENT	1.05E-09	990	194	17	1.717172
8	7517	MUSCLE_ORGAN_DEVELOPMENT	1.43E-07	407	194	10	2.457002
20	43005	NEURON_PROJECTION	6.85E-08	1317	64	11	0.835232
20	45202	SYNAPSE	5.74E-15	1332	64	17	1.276276
20	7269	NEUROTRANSMITTER_SECRETION	2.17E-08	168	64	6	3.571429
31	33002	MUSCLE_CELL_PROLIFERATION	1.01E-07	241	28	5	2.074689
31	35265	ORGAN_GROWTH	2.76E-06	203	28	4	1.970443
High 0 h and 1 day							
10	43043	PEPTIDE_BIOSYNTHETIC_PROCESS	2.94E-91	736	159	73	9.918478
10	6413	TRANSLATIONAL_INITIATION	6.11E-111	192	159	62	32.29167
12	6396	RNA_PROCESSING	1.66E-10	917	131	16	1.74482
High 0 h and 3–7 day							
15	5768	ENDOSOME	1.10E-06	918	92	11	1.198257
21	70160	TIGHT_JUNCTION	0.0000079 6	126	47	4	3.174603
21	5912	ADHERENS_JUNCTION	0.0000238	169	47	4	2.366864
21	43297	APICAL_JUNCTION_ASSEMBLY	3.57E-05	64	47	3	4.6875
21	2520	IMMUNE_SYSTEM_DEVELOPMENT	4.65E-05	990	47	7	0.707071
26	42582	AZUROPHIL_GRANULE	3.56E-06	155	36	4	2.580645
26	46903	SECRETION	1.02E-05	1680	36	8	0.47619
26	33043	REGULATION_OF_ORGANELLE_ORGANIZATION	1.59E-05	1267	36	7	0.552486
21	7010	CYTOSKELETON_ORGANIZATION	4.03E-05	1335	47	8	0.599251
28	44257	CELLULAR_PROTEIN_CATABOLIC_PROCESS	1.49E-06	782	34	7	0.895141
High 0 h and 7 day							
2	7049	CELL_CYCLE	2.09E-32	1864	824	110	5.901288
2	51254	POSITIVE_REGULATION_OF_RNA_METABOLIC_PROCESS	9.94E-36	1710	824	110	6.432749
2	51276	CHROMOSOME_ORGANIZATION	9.45E-33	1223	824	89	7.277187
2	15630	MICROTUBULE_CYTOSKELETON	1.76E-22	1220	824	74	6.065574
24	2521	LEUKOCYTE_DIFFERENTIATION	7.01E-14	520	44	11	2.115385
24	2682	REGULATION_OF_IMMUNE_SYSTEM_PROCESS	3.44E-12	1670	44	14	0.838323
24	46649	LYMPHOCYTE_ACTIVATION	8.65E-14	736	44	12	1.630435

GO terms of interest were chosen to highlight the various biological processes occurring at each time-point

**Table 2:** **GO term enrichment annotations for ED18 enteroid network clusters grouped into similar time-point expression profiles.**

Cluster	GO term no	Significantly-enriched GO term (*p* < 0.05)	*p* value	Genes in gene set	Genes in cluster	Genes in overlap	% overlap
High 1 day							
3	6091	GENERATION_OF_PRECURSOR_METABOLITES_AND_ENERGY	9.68E-59	532	704	81	15.22556
3	46034	ATP_METABOLIC_PROCESS	9.19E-56	303	704	64	21.12211
3	7005	MITOCHONDRIAL_ORGANIZATION	4.17E-71	528	704	91	17.23485
19	98798	MITOCHONDRIAL_PROTEIN_COMPLEX	2.19E-08	260	69	7	2.692308
19	16071	MRNA_METABOLIC_PROCESS	2.65E-09	840	69	11	1.309524
High 1–7 day							
9	46903	SECRETION	1.32E-08	1680	179	24	1.428571
9	5773	VACUOLE	7.76E-10	781	179	18	2.304738
9	44257	CELLULAR_PROTEIN_CATABOLIC_PROCESS	5.64E-09	782	179	17	2.173913
11	70727	CELLULAR_MACROMOLECULE_LOCALIZATION	2.49E-07	1930	143	20	1.036269
11	6508	PROTEOLYSIS	6.74E-08	1779	143	20	1.124227
13	44255	CELLULAR_LIPID_METABOLIC_PROCESS	2.80E-07	1064	109	14	1.315789
13	16050	VESICLE_ORGANIZATION	2.97E-11	330	109	12	3.636364
14	30199	COLLAGEN_FIBRIL_ORGANIZATION	4.24E-06	55	100	4	7.272727
14	43062	EXTRACELLULAR_STRUCTURE_ORGANIZATION	4.99E-08	373	100	9	2.412869
High 1–4 day							
4	44281	SMALL_MOLECULE_METABOLIC_PROCESS	1.06E-22	1962	658	83	4.230377
4	44257	CELLULAR_PROTEIN_CATABOLIC_PROCESS	9.84E-23	782	658	52	6.649616
4	6886	INTRACELLULAR_PROTEIN_TRANSPORT	7.04E-29	1151	658	71	6.168549
6	6629	LIPID_METABOLIC_PROCESS	4.53E-13	1405	355	38	2.704626
6	7049	CELL_CYCLE	3.37E-11	1864	355	41	2.199571
High 3–7 day							
16	34378	CHYLOMICRON_ASSEMBLY	4.54E-07	10	88	3	30
16	42304	REGULATION_OF_FATTY_ACID_BIOSYNTHETIC_PROCESS	1.99E-06	56	88	4	7.142857
16	46486	GLYCEROLIPID_METABOLIC_PROCESS	4.25E-06	414	88	7	1.690821
18	6887	EXOCYTOSIS	2.29E-06	900	69	10	1.111111
18	8047	ENZYME_ACTIVATOR_ACTIVITY	2.56E-05	520	69	7	1.346154
18	46903	SECRETION	1.87E-05	1680	69	12	0.714286
High 7 day							
5	30030	CELL_PROJECTION_ORGANIZATION	3.09E-29	1545	491	72	4.660194
5	45595	REGULATION_OF_CELL_DIFFERENTIATION	2.03E-23	1881	491	71	3.774588
5	35295	TUBE_DEVELOPMENT	4.70E-18	1122	491	48	4.278075
5	22008	NEUROGENESIS	1.95E-29	1625	491	74	4.553846
7	31012	EXTRACELLULAR_MATRIX	1.55E-17	531	320	28	5.27307
7	48870	CELL_MOTILITY	1.75E-13	1758	320	42	2.389078
7	48514	BLOOD_VESSEL_MORPHOGENESIS	5.18E-13	686	320	26	3.790087
7	46695	REGULATION_OF_CELL_DIFFERENTIATION	5.59E-14	1163	320	38	3.267412
7	30030	CELL_PROJECTION_ORGANIZATION	5.59E-14	1545	320	40	2.588997
7	22008	NEUROGENESIS	5.12E-16	1625	320	44	2.707692
7	22610	BIOLOGICAL_ADHESION	7.11E-19	1425	320	45	3.157895
7	1816	CYTOKINE_PRODUCTION	3.72E-12	808	76	12	1.485149
7	2521	LEUKOCYTE_DIFFERENTIATION	1.83E-08	520	76	8	1.538462

GO terms of interest were chosen to highlight the various biological processes occurring at each time-point.

**Figure 4:**
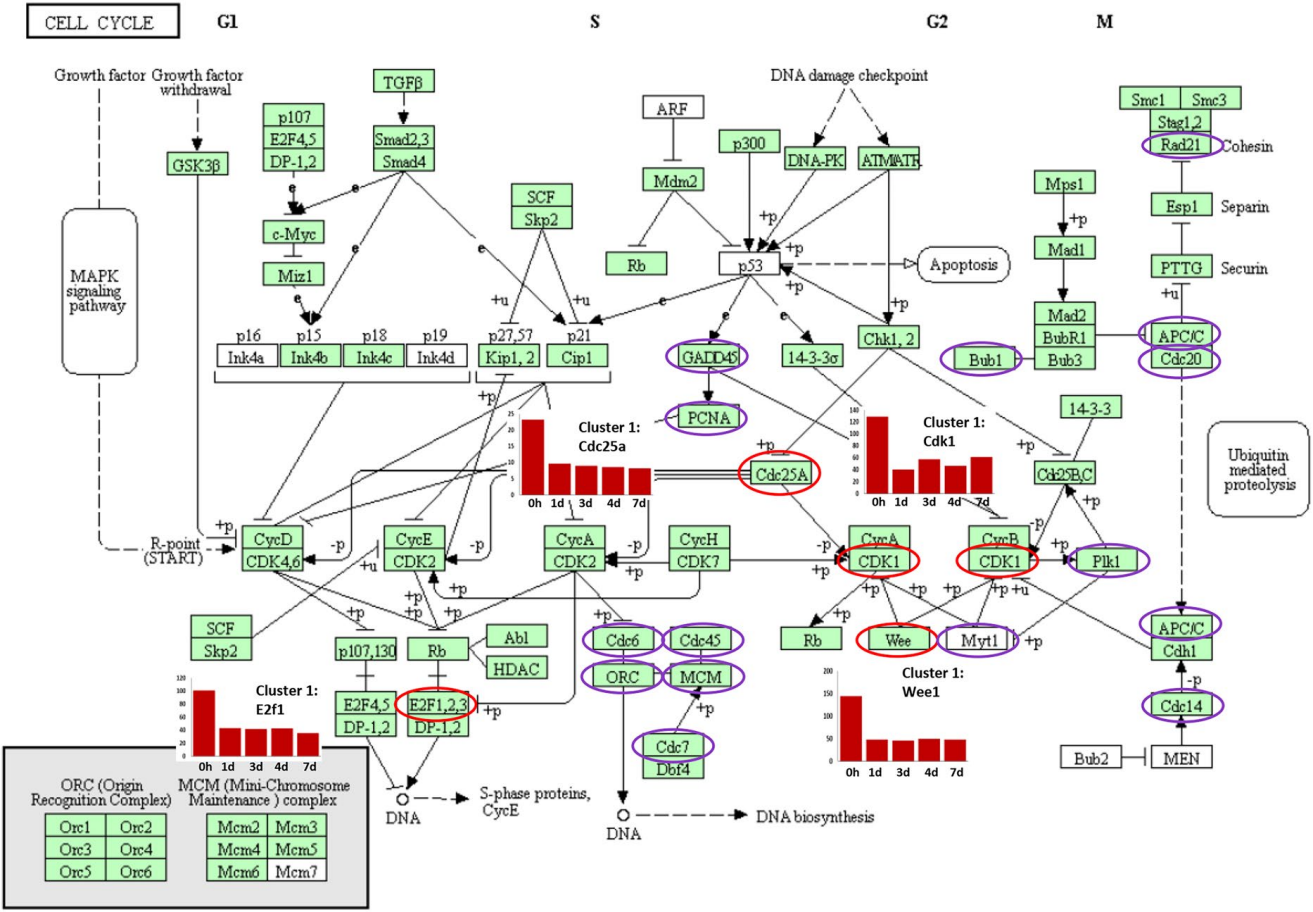
**Cell cycle related genes highlighted in the ED18 intestinal villi**. KEGG pathway: Cell cycle *Gallus gallus* (chicken). Purple and red circled genes were contained in embryonic tissue analysis Cluster 1 whose mean gene expression profile was high in ED18 intestinal villi compared to enteroids. Associated GO terms in Cluster 1 were predominantly expressed in the cell cycle. Red circled genes have their mean expression profiles demonstrated in adjacent graphs. The graph x axis shows the samples ordered from time of cultures with 0 h representing isolated ED18 intestinal villi. The *y* axis shows the mean expression intensity for the cluster (transcripts/million reads, TPM).

The 0 h time point (freshly isolated villi) denotes a stage of rapid development in the late embryonic intestine and accordingly GO terms associated with tissue development were predominant in the villus-related clusters including regulation of cell differentiation, organ growth and cell cycle (Table 1). Analysing cell cycle related cluster 1 in more detail, multiple individual genes were also represented in the KEGG *Gallus gallus* cell cycle pathway map e.g. cyclin dependent kinase 1 (*CDK1*), *WEE1*, cell division cycle 25A (*CDC25A*), eukaryotic translation termination factor 1 (*ERF1*) (Figure 4, Additional file 4). GO terms associated with cell cycle were also found in 1–4 day enteroid clusters (Table 2). In addition, many of the genes enriched in 0 h and 1 day enteroids were also represented in GO terms associated with transcription and translational regulation including translational initiation, peptide biosynthetic process and RNA processing (Table 1).

Cluster 3, which was highly expressed in 1 day enteroids, contained genes with GO terms associated with cell energy production such as generation of precursor metabolites and energy and adenosine triphosphate (ATP) metabolic process (Table 2). This transcriptional analysis, confirms as anticipated, that cell proliferation is especially prevalent in the first couple of days of enteroid cultivation, a timeframe also reflective of the rapid development of the post-hatch chick intestine in vivo. Consistent with the transcriptional analysis, EdU + staining of enteroid cultures showed the percentage of proliferating cells dropped from almost 10% at 14 h to 5% after 7 days of culture (Additional file 7, [16]).

### Enteroid cultures suggest prolonged expression of intestinal stem cell homeostasis pathways

**Figure 5:**
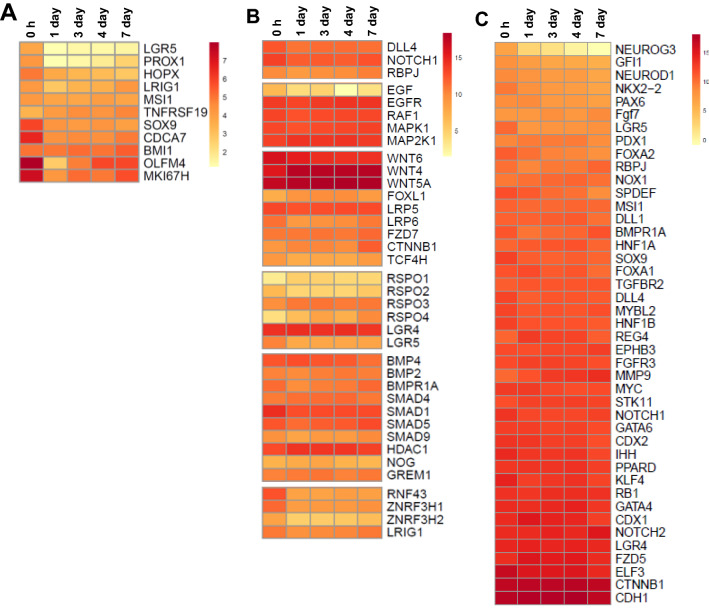
**Single gene analyses related to stem cell homeostasis and epithelial cell differentiation**. Expression of **A** stem cell markers, **B** Wnt signalling pathway genes, and **C** intestinal epithelial cell differentiation related genes at 0 h and enteroids at 1, 3, 4 and 7 days of culture were analysed by RNA sequencing. Heat maps show the mean relative expression levels (log2 counts per million reads) of a range of mammalian gene-sets. RNA sequencing data is representative of 3 independent experiments, each comprising of 2 technical replicates, each containing 3 embryos.

Intestinal stem cell (ISC) maintenance is essential for enteroid cultures and is largely dependent on the Wingless-related integration site (WNT) signalling pathway. Immediately after isolation of the villi, the expression of some ISC-related genes was higher compared to 1 day enteroids most likely reflecting the relative abundance of ISC in ED18 villi (Figure 5A, Additional file 8). In addition, many ISC and WNT signalling pathway genes were steadily expressed in the enteroids including *WNT4* and *WNT5a,* both of which are expressed by mesenchymal cells in mice (Figure 5B, Additional file 8, [23]). There was also a 7–17 fold downregulation of negative WNT regulators e.g. Zinc and ring finger 3 H1 (*ZNRF3H1), ZNRF3H2,* Ring finger protein 43 (*RNF43*) in the enteroids compared to 0 h (Figures 5B). However, several ISC markers such as *OLFM4* and homeostatic factors such as *WNT6*, typically produced by Paneth cells in murine crypts [24], were significantly downregulated in the enteroids compared to 0 h (Figures 5A and B; Additional file 9). This reduced expression most likely reflects a dilution of the villi stem cell population as the enteroids are maturing in culture but could also suggest a reduction in activity. However, the expression of most of these ISC genes and related homeostatic factors subsequently remained stable throughout the later culture time points.

### Enteroid cultures reproduce the cellular diversity of the intestinal epithelium

Expression of gene sets characteristically associated with mammalian Paneth cells, goblet cells, enterocytes, enteroendocrine, transit amplifying, intestinal stem, and tuft cells were identified in the enteroids throughout the culture period. Furthermore, the transcriptomes for most epithelial cell-types remained relatively stable during this period. However, there was a reduction in gene expression associated with differentiation to enteroendocrine cells including neurogenin 3 (*NEUROG3*; 15-fold) and forkhead box A2 (*FOXA2*; two-fold) (Figure 5C)*.* In addition, the expression of the epithelial differentiation marker alkaline phosphatase (*ALPP*) decreased over time. In summary, the transcriptome analysis suggests that the cellular diversity in the enteroid cultures is similar to the embryonic intestine.

### Brush border develops in the enteroid cultures

**Figure 6:**
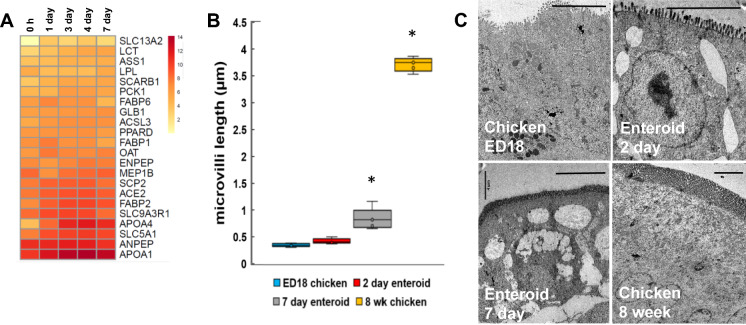
**Single gene analyses of development and function of the enteroid cultures**. Expression of **A** digestion related genes in ED18 intestinal villi (0 h) and enteroids at 1, 3, 4 and 7 days of culture were analysed by RNA sequencing. Heat maps show the mean relative expression levels (log_2_ counts per million reads) of a range of mammalian gene-sets. RNA sequencing data is representative of 3 independent experiments, each comprising of 2 technical replicates, each containing 3 embryos**.** **B** Quantification of microvilli length in ED18 intestine, 3 and 7 day enteroids and 8 week chicken intestine measured from TEM images; averaged for 3 cultures containing 2–3 embryos. *P < 0.05, unpaired Student’s *t*-test compared to ED18. **C** Representative TEM images demonstrating microvilli length in ED18 intestine, 2 and 7 day chicken enteroids, and 8 week chicken intestine. Scale bar: 4 µm.

In order to confirm if this in vitro intestinal model represented the typical array of in vivo functions, the transcriptome was also investigated for evidence of absorption and digestion at the gene level. The gene expression clusters that were predominantly expressed in the enteroids data sets compared to 0 h (freshly isolated villi) were significantly enriched in genes with GO terms related to cellular lipid metabolic process, chylomicron assembly, cellular protein catabolic process and glycerolipid metabolic process (Table 2). Looking at small intestinal digestion related genes in the enteroids in further detail, there was a relatively steady expression of many genes over the sample time points including brush border enzymes e.g. lactase (*LCT*) and solute carrier family *SLC13A1*, and those involved in fat metabolism e.g. apolipoprotein *APOA1* and *APOA4* (lipid transport), fatty acid binding protein *FABP2* (fatty acid transport), and sterol carrier protein *SCP2* (cholesterol uptake and transport) (Figure 6A) [25, 26].

The expression of genes related to the GO term “cell projection organisation” in the 7 day enteroids may reflect microvilli development, essential structures which increase the intestinal epithelial surface area for both secretion and absorption. To confirm whether the microvilli of apical-out enteroids continue to develop in vitro, their lengths across the culture period were measured in transmission electron microscopy images. This analysis revealed that the microvilli on the enterocyte surfaces doubled in the enteroids after 7 days of culture when compared to the ED18 intestine (Figures 6B and C). This morphological analysis alongside the transcriptome data suggests that the enteroids develop post-hatch digestive functions during cultivation.

### Development of a comprehensive lamina propria component

**Figure 7:**
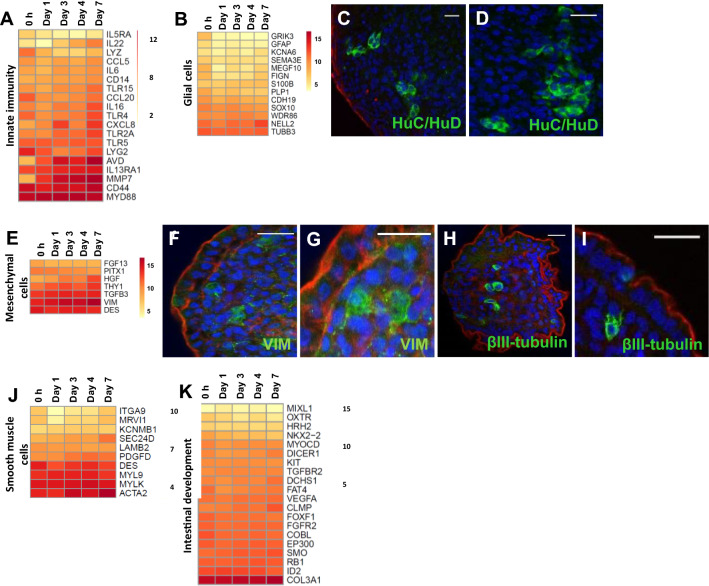
**Cell types identified in the chicken enteroid lamina propria**. Expression of **A** innate immune function related genes, **B** glial cell, **E** mesenchymal cell, **J** smooth muscle genes and **K** intestinal development gene sets in freshly isolated villi (0 h), 1, 3, 4 and 7 day chicken enteroids was compared by RNA sequencing analysis. Heat maps show the mean relative expression levels (log_2_ counts per million reads) of a range of mammalian gene-sets. RNA sequencing data is representative of 3 independent experiments, each comprising of 2 technical replicates, each containing 3 embryos. **C**–**D**, **F**–**I** Confocal images of chicken enteroids stained for lamina propria cell population markers (green) and counterstained with DAPI (blue) and Phalloidin (red). Enteroids at 2 days of culture stained for (**C**,** D**) HuC/HuD (green, glial cells), (**F**, **G**) vimentin (green, mesenchymal cells), (**H**, **I**) βIII-tubulin (green, mesenchymal cells). Scale bar: 20 µm. Images are representative of data from at least 3 independent cultures each containing 2–3 embryos.

ED18 villi isolation and floating culture conditions resulted in an apical-out enteroid phenotype with a lamina propria core [16]. In vivo the lamina propria is composed of multiple elements including immunologically competent cells, components of the enteric nervous system (glial cells and neuron axons) and mesenchymal cells. Consistent with this, GO terms related to immune system development and function were represented during the entirety of the culture period (Tables 1 and 2). Genes representative of several populations of lymphocytes, including T cells, B cells, macrophages, dendritic cells and NK cells were present at all time points (Additional file 10), consistent with the detection of CD45 + lymphocytes in the enteroids (Additional file 10). Genes associated with innate immune function were also relatively stable expressed over time, although some genes increased and some genes decreased over time in culture (Figure 7A; Additional file 9), e.g. pattern recognition receptors (Toll-like receptor 3 (*TLR3*) increased, whereas cytokine receptor gene *IL20RB* decreased, *IL1B* expression varied over time and antimicrobial enzyme avidin (*AVD*) increased.

Development of both the enteric nervous system and the muscularis mucosa was indicated in the 0 h transcriptomes with the expression of many genes with GO terms related to neuron projection, synapse and muscle cell proliferation, as would be expected for this late stage of embryonic maturation (Table 1). Further analysis of the genes within villi-associated cluster 20 confirmed the presence of many genes encoding proteins associated with synaptic signalling, neurotransmission, neuron growth as well as several genes encoding ligand- or voltage-gated ion channels (Additional File 1, cluster 20)*.* Glial cell marker genes displayed steady expression in the enteroids (Figure 7B) and the presence of the enteric nervous system was further confirmed by staining for HuC/HuD, a pan-neuronal marker which labels neurons and glial cells. Multiple HuC/HuD + cells were identified scattered individually (Figure 7C) or in clusters (Figure 7D) throughout the central core of the 2 day enteroids. Since the presence of neuronal cell bodies would not be expected in the lamina propria, these are likely glial populations, reflecting characteristic cellular and tissue spatial distribution patterns [27].

Vimentin is the major cytoskeletal component of mesenchymal cells and, alongside other mesenchymal markers and highly expressed in the enteroid cultures (Figure 7E). Vimentin positive stained cells were accordingly restricted to the enteroid lamina propria core (Figures 7F and G). βIII-tubulin is generally used as a neuronal differentiation marker but it has also been shown to be expressed by other cell types including mesenchymal stem cells and perivascular cells [28, 29]. Since gut neuronal bodies are located in the submucosal ganglia [30], the scattered distribution of βIII-tubulin + cells in the enteroid core are most likely representative of mesenchymal stem cells with typical perinuclear staining (Figures 7H and I).

Smooth muscle fibres, blood and lymph vessels are typically present within a villus core (Figure 7J). GO terms related to blood vessel morphogenesis and epithelial or endothelial tube development were highly enriched on day 7 of culture (Table 2). A major contributor to angiogenesis, vasculogenesis and endothelial cell growth is vascular endothelial growth factor A (*VEGFA*), which is produced by many cell types including macrophages, and its transcript levels were steadily expressed over the enteroid cultures (Figure 7K). Analysis of genes specifically related to intestinal development showed many have steady transcript levels through the enteroid cultures (Figure 7K) [26]. This work provides a thorough transcriptomics validation of the enteroid core and provides a foundation for future studies to determine the full diversity of enteroid cell populations on a transcriptomic, proteomic and functional level.

### No indication of epithelial-mesenchymal transition in 7 days enteroids

**Figure 8:**
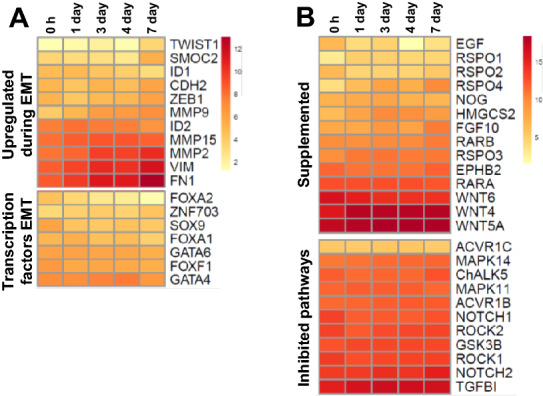
**EMT and growth factor single gene analysis of villi and enteroid cultures**. Expression of **A** EMT and **B** growth factor associated genes in ED18 intestinal villi (0 h) and enteroids analysed by RNA sequencing. Heat maps show the mean relative expression levels (log2 counts per million reads) of a range of mammalian described gene-sets. RNA sequencing data is representative of 3 independent experiments, each comprising of 2 technical replicates, each containing 3 embryos.

Although the floating chicken enteroid cultures develop profuse villus-like structures without external supplementation, they are not viable long-term. We previously showed these enteroid cultures display no general increase in cell stress-related genes indicating that the culture conditions are not causing excessive apoptosis or necrosis [16]. Therefore a possible cause of the reduced enteroid longevity could be due to a change in cell type and function. During epithelial-mesenchymal transition (EMT), epithelial cells acquire mesenchymal fibroblast-like properties, losing apical-basal polarity, weakening cell–cell junctions, rearranging the cytoskeleton and acquiring increased motility (reviewed in [31]). EMT will occur in the basal-out enteroids of some mammalian species unless there is specific inhibition of transforming growth factor β (TGFβ) and p38 mitogen activated protein kinase (MAPK) pathways to block this activity. We therefore compared the differentially expressed genes (FDR < 0.05, minimum logFC > 1) in 7 day versus 4 day enteroids to determine whether there was significant upregulation of genes associated with EMT and epithelial migration. This analysis showed a ~six-fold increase in fibroblast activation protein (*FAP*), hepatocyte growth factor (*HGF*) and integrin subunit A4 (*ITGA4*) (Additional file 9). Analysis of genes generally upregulated during EMT showed a steady expression across the enteroid time points and although some were significant differentially expressed the fold changes between time points were low (Figure 8A and Additional file 9). A similar steady expression was found of EMT-associated transcription factors (Figure 8A, Additional file 9), and evidence of ongoing epithelial development, including microvilli lengthening, and digestive function was still apparent at 7 days of culture (Figures 5C and 7A–C). This suggests that it is unlikely that the lack of long term viability and ability to passage the enteroids is mediated by EMT.

To ascertain if deficiencies in supplemented growth factors and/or inhibited molecules, typically targeted in mammalian cultures, could be the cause of reduced culture longevity, the expression of genes encoding key factors was compared between 0 h (freshly isolated villi) and chicken enteroids (Figure 8B). Most genes had consistent to increasing expression across the cultures except for epidermal growth factor (*EGF*), *WNT6* and R-spondin 2 (*RSPO2*) which were downregulated in the enteroids compared to 0 h. Of note, *MAPK14* and *MAPK11* which are targeted by p38MAPK-inhibitors, and *TGFB1* and Rho associated coiled-coil containing protein kinase 1 (*ROCK1*) which are targeted by TGFβ-inhibitors were also steadily expressed. In summary, both EMT and growth factor deficiencies are unlikely contributors to the inability to passage apical-out enteroids, and further studies are required to ascertain both the cause and potential solutions to alleviate this longevity problem.

## Discussion

Transcriptome profiling analyses were performed on data from floating avian enteroid and ED18 intestinal villi (0 h) samples to demonstrate the reproducibility of gene expression within and between cultures, show the maturation of embryonic enteroids to post-hatch functions in culture, and explore the propagation issues facing apical-out enteroid cultures.

To support reproducibility of cell based experiments, a desirable trait for any tissue culture model is culture consistency. This is of particular importance in multicellular complex organoid models. The enteroid RNA-seq analysis demonstrated that the main cause for differences between sample transcriptomes was time of culture, with a high degree of similarity in global transcriptional activity between technical and biological replicates. As would be expected of biologically distinct samples with natural variation, there were slight differences in biological replicate transcriptomes identified within the PCA [32]. Individual variability in donor genotype, microbiome and disease status has been shown to affect the epithelial phenotype of enteroid cultures [33–35]. Compared to wild chicken breeds, commercial layer chicken lines have a fairly low genotypic diversity therefore the effect of variability of genotype on the Hy-Line chicken enteroid cultures should be small [36]. The use of late-embryonic villi to develop the enteroid cultures also reduces the chance that infection of the donor will influence the culture phenotype, although some avian viruses can be vertically transmitted [37]. The influence of microbiome on cultures will be limited since the avian intestine is mostly colonised post-hatch, enabling the use of enteroids for microbiome-host interaction studies. However, ensuring adequate numbers of biological replicates for enteroid experiments will improve the efficiency of statistical testing.

The divergence of ED18 intestinal villi and enteroid samples to opposite sides of the PC1 axis highlights a marked difference in their global gene expression profiles. This disparity may in part be due to adaptions to the in vitro culture environment, which do not reproduce all in vivo factors that affect intestinal development and maturation, including a developing microbiome. In addition, it may in part be due to the dramatic and rapid development of budding enteroids from embryonic villi. In the chicken during the first 24 h post-hatch there are striking changes in the mucosal morphology of the small intestine, including intensive cryptogenesis and a dramatic increase in villus length [10, 38]. Similar morphological changes are demonstrated by the growing chicken enteroids where there is rapid closure of the villi fragments within hours of plating out and development of multiple buds by 1 day which continue to lengthen over the following days [16]. In conjunction with the rapid growth, the 1 day enteroid transcriptome denotes upregulation of many genes involved in development, proliferation and differentiation. The reproducibility of in vitro models is important for a robust analysis, and the enteroids display steady transcriptional profiles for cell differentiation and most ISC homeostasis gene sets in the first 7 days of culture, with 3–4 days highlighted as a particularly stable timeframe [39]. However, although steady expression during the culture period was observed, there is a substantial decrease in gene expression of ISC genes when freshly isolated villi are compared with cultured enteroids. Whether this is due to the isolation step, dilution of the gene expression during proliferation or decrease of stem cells needs further investigation. Zhang and Wong [40] also demonstrated an increase in OLFM4 expression in post-hatch chicks over 0–7 days of age indicate this ISC marker may play a role in intestinal maturation.

As a precocial species, in parallel with the morphological changes, the ability of the chick gut to digest and absorb nutrients has been shown to increase quickly enabling chickens to have an almost fully mature intestine shortly after hatch [11, 38, 41]. For example, in vivo brush border enzyme activities have been shown to increase rapidly in the post-hatch chicken and the enteroids mirror this with an increase in expression of selected brush border enzyme genes [38]. Nutrient digestion and absorption in the enteroids was significantly centered in the lipids but not the other macro-nutrients. This mirrors the post-hatch chick intestine where there is a selective hierarchy at this age for fatty acid uptake over glucose or amino acids due to the yolk being an important source of nutrients for up to 72 h post-hatch [42, 43]. Although transcript differences do not necessarily equate with functional significance, that the chicken enteroids develop strong expression of digestion-related genes and associated pathways strongly indicates that the in vitro conditions contain the important cues for post-hatch maturation and correspond to post hatch transcriptomic analysis of the small intestine [44, 45].

Despite the capability existing to consecutively passage gel-embedded chicken enterospheres (organoid nomenclature for the poorly-differentiated spherical structures observed using this technique), currently there is no evidence for the ability to passage floating enteroid cultures [13, 14, 16, 46, 47]. Various trials have been performed in the floating chicken enteroids, but continued bud growth after passage has not been recognised [16]. Blockade of p38MAPK and TGF-β pathways in human, bovine and porcine gel-embedded enteroid cultures is required, in part, to help to protect the cells within them against EMT [4, 48–50]. However, trials of p38MAPK and TGF-β molecular inhibitors in floating chicken enteroid media showed no improvement in post-passage growth or budding [16]. Murine enteroid cultures require active supplementation with TGF-β in order to induce an EMT model indicating species-specific differences in these pathways which may also be present in the chicken [51].

Using transcriptome-wide comparisons of avian apical-out enteroid and ED18 intestinal villi samples we have constructed a detailed picture of gene expression patterns in developing chicken enteroids and the late-embryonic chicken intestine. This has demonstrated the consistency of this culture model as well as identifying hallmarks of multiple key intestinal functional attributes such as to development of digestive function and organ development. The enteroids have been demonstrated to have a transcriptionally stable timeframe where they are morphologically mature, indicating useful culture stages to conduct physiologically-relevant experiments. Although the enteroids do not have the ability to be propagated, these results indicate that this unique model is an adequate in vitro tool for post-hatch chicken intestinal studies into e.g. host–pathogen interactions, growth of previously unculturable micro-organisms, breed phenotype and nutrition studies, and feed additive and heat stress trials (reviewed in [52]). Introduction of the apical-out chicken enteroid and ED18 chicken intestinal villi transcriptome to the wider scientific community will provide a valuable resource for future avian intestinal research.

## Supplementary Information

**Additional file 1. RNA samples collected for the transcriptome sequencing. **Description of the ED18 intestinal villi and chicken enteroid RNA samples submitted for RNA sequencing.

**Additional file 2. Primers and probes used in RT-qPCR.**

**Additional file 3. Primary antibodies used for immunohistochemistry.**

**Additional file 4. Genes found in the largest 50 co-expression cluster derived from the RNA-seq analysis data**. Table providing list of genes found in each co-expression cluster in the network graph derived from the RNA-seq analysis data.

**Additional file 5. Mean expression profiles of the genes in each of the largest 50 co-expression clusters. **Individual mean expression profiles of the genes in each of the largest 50 co-expression clusters derived from the network graph. The *x* axis shows the samples ordered by time of cultures. The *y* axis shows the mean expression intensity (transcripts/million reads, TPM) for the cluster.

**Additional file 6. GO term enrichment annotations for the genes in the largest 50 co-expression clusters. **Table listing the representative GO term enrichment annotations for the genes in the largest 50 co-expression clusters derived from the network graph. Note, the Entrez Gene IDs are for human orthologs.

**Additional file 7. Proliferation in the enteroids. **Graph demonstrating the percentage of proliferating (EdU+) cells within enteroids over 7 days of culture, alongside z-stack image of proliferating (EdU+) cells within a 14 h enteroid.

**Additional file 8. Cell-type gene expression in chicken enteroid cultures. **Expression of stem cell, Paneth cell, enteroendocrine cell, goblet cell and enterocyte genes in 1 day chicken enteroid cultures. Confocal images of chicken enteroids at 2 days of culture stained for.

**Additional file 9. Differentially expressed gene summary. **Table listing the DEGs (logFC, *P*-value, FDR) from adjacent culture time points.

**Additional file 10. Leucocytes identified in the chicken enteroid lamina propria.** Expression of immune cell population gene sets analysed by RNA sequencing analysis. Confocal images of chicken enteroids at 2 days of culture stained for CD45.

## Data Availability

The mRNA expression datasets for this study have been deposited in the European Nucleotide Archive (ENA) at EMBL-EBI under accession number PRJEB37491 and PRJEB51227. The datasets used and/or analysed during the current study are available from the corresponding author on reasonable request.
