# Supplementary material for: Temporal transcriptome profiling of floating apical out chicken enteroids suggest stability and reproducibility
Source: Vet Res. 2023 Feb 15;54:12. doi: 10.1186/s13567-023-01144-2 (PMC9933378; doi:10.1186/s13567-023-01144-2)
Supplement: Supplementary file 1 — Additional file 1. RNA samples collected for the transcriptome sequencing. Description of the ED18 intestinal villi and chicken enteroid RNA samples submitted for RNA sequencing. [file 13567_2023_1144_MOESM1_ESM.docx]

**Additional File 1. RNA samples collected for the transcriptome sequencing.** Description of the ED18 intestinal villi and chicken enteroid RNA samples submitted for RNA sequencing with details of quality (RIN), concentration (both obtained by tapestation) and raw reads (obtained by NovaSeq S2 100PE). *(*Gallus gallus*)

| **Samples** | **Samples description** | **Biological**  **replicate** | **Technical**  **replicate** | **RIN** | **Concentration**  **(ng/μL)** | **Raw reads**  **(×10^8^)** |
| --- | --- | --- | --- | --- | --- | --- |
| COA_1_0h | Chicken* villi  0 h | 1 | 1 | 9.1 | 345.8 | 1.83 |
| COA_2_0h | Chicken* villi  0 h | 1 | 2 | 9.2 | 327.6 | 1.62 |
| COA_1_24h | Chicken* enteroids 1 day culture | 1 | 1 | 6.1 | 153.4 | 1.42 |
| COA_2_24h | Chicken* enteroids 1 day culture | 1 | 2 | 6.1 | 212.55 | 1.68 |
| COA_1_72h | Chicken* enteroids 3 day culture | 1 | 1 | 7.7 | 107.25 | 1.68 |
| COA_2_72h | Chicken* enteroids 3 day culture | 1 | 2 | 8.1 | 198.25 | 1.47 |
| COA_1_96h | Chicken* enteroids 4 day culture | 1 | 1 | 8.6 | 135.85 | 1.65 |
| COA_2_96h | Chicken* enteroids 4 day culture | 1 | 2 | 7.3 | 291.85 | 1.51 |
| COA_1_168h | Chicken* enteroids 7 day culture | 1 | 1 | 9.5 | 260.65 | 1.59 |
| COA_2_168h | Chicken* enteroids 7 day culture | 1 | 2 | 9.3 | 375.05 | 1.79 |

| **Samples** | **Samples description** | **Biological**  **replicate** | **Technical**  **replicate** | **RIN** | **Concentration**  **(ng/μL)** | **Raw reads**  **(×10^8^)** |
| --- | --- | --- | --- | --- | --- | --- |
| COB_1_0h | Chicken* villi  0 h | 2 | 1 | 9 | 421.2 | 1.54 |
| COB_2_0h | Chicken* villi  0 hr | 2 | 2 | 8.8 | 427.05 | 1.56 |
| COB_1_24h | Chicken* enteroids 1 day culture | 2 | 1 | 6.7 | 378.3 | 1.58 |
| COB_2_24h | Chicken* enteroids 1 day culture | 2 | 2 | 6.4 | 208 | 1.47 |
| COB_1_72h | Chicken* enteroids 3 day culture | 2 | 1 | 7.9 | 107.25 | 1.45 |
| COB_2_72h | Chicken* enteroids 3 day culture | 2 | 2 | 7.2 | 343.2 | 1.5 |
| COB_1_96h | Chicken* enteroids 4 day culture | 2 | 1 | 7.7 | 260.65 | 1.62 |
| COB_2_96h | Chicken* enteroids 4 day culture | 2 | 2 | 8.1 | 234.59 | 1.5 |
| COB_1_168h | Chicken* enteroids 7 day culture | 2 | 1 | 9.6 | 274.3 | 1.65 |

| **Samples** | **Samples description** | **Biological**  **replicate** | **Technical**  **replicate** | **RIN** | **Concentration**  **(ng/μL)** | **Raw reads**  **(×10^8^)** |
| --- | --- | --- | --- | --- | --- | --- |
| COC_1_0h | Chicken* villi  0 h | 3 | 1 | 9.2 | 298.35 | 1.6 |
| COC_2_0h | Chicken* villi  0 h | 3 | 2 | 9.1 | 283.64 | 2.15 |
| COC_1_24h | Chicken* enteroids 1 day culture | 3 | 1 | 7.5 | 156.65 | 1.33 |
| COC_2_24h | Chicken* enteroids 1 day culture | 3 | 2 | 7.6 | 244.4 | 1.67 |
| COC_1_72h | Chicken* enteroids 3 day culture | 3 | 1 | 7.9 | 178.1 | 1.47 |
| COC_2_72h | Chicken* enteroids 3 day culture | 3 | 2 | 8.6 | 269.75 | 1.95 |
| COC_1_96h | Chicken* enteroids 4 day culture | 3 | 1 | 8.9 | 104.65 | 1.55 |
| COC_2_96h | Chicken* enteroids 4 day culture | 3 | 2 | 8.9 | 148.2 | 1.81 |
| COC_1_168h | Chicken* enteroids 7 day culture | 3 | 1 | 9.8 | 257.4 | 1.82 |
| COC_2_168h | Chicken* enteroids 7 day culture | 3 | 2 | 9.5 | 396.5 | 1.46 |
