# Supplementary figures and images for: Temporal transcriptome profiling of floating apical out chicken enteroids suggest stability and reproducibility
Source: Vet Res. 2023 Feb 15;54:12. doi: 10.1186/s13567-023-01144-2 (PMC9933378; doi:10.1186/s13567-023-01144-2)

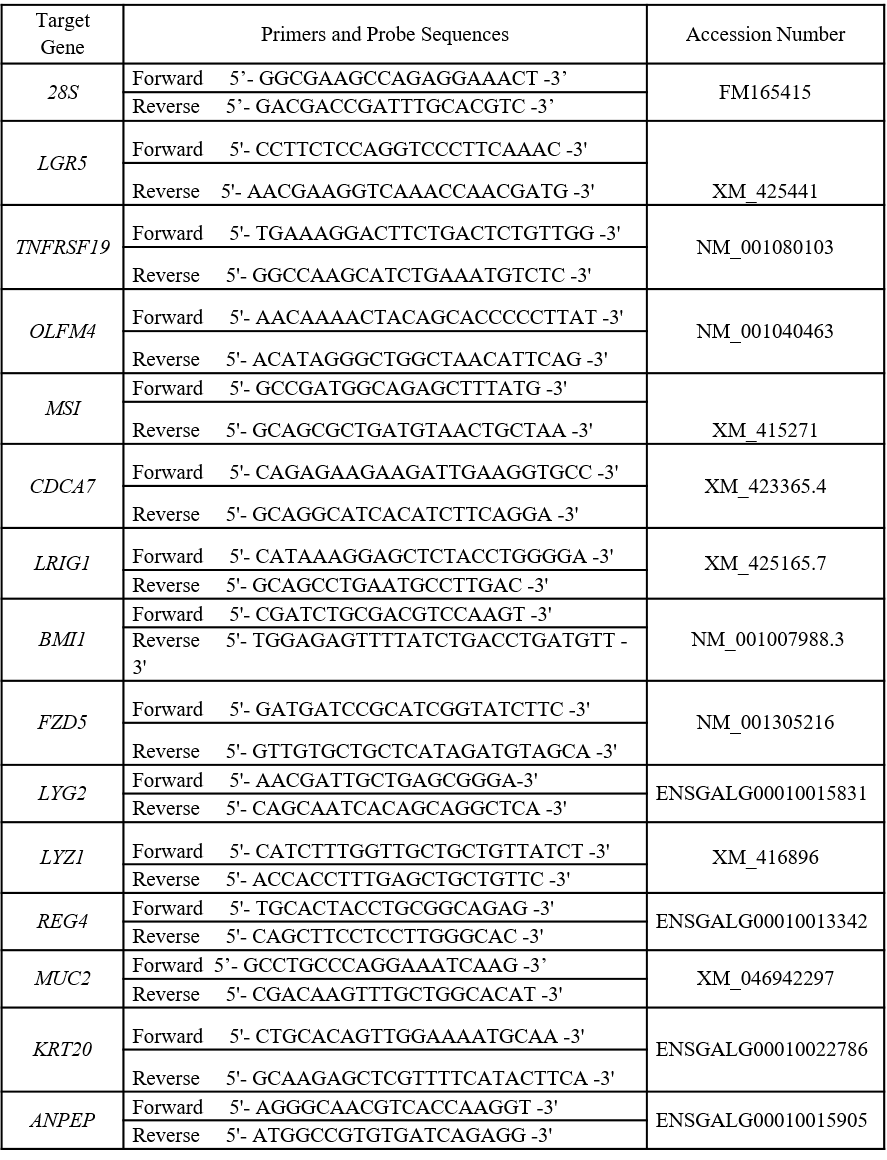


**Additional file 2.** Primers and probes used in RT-qPCR

Supplement: Supplementary file 2 — Additional file 2. Primers and probes used in RT-qPCR. [file 13567_2023_1144_MOESM2_ESM.docx]
