# Supplementary material for: Temporal transcriptome profiling of floating apical out chicken enteroids suggest stability and reproducibility
Source: Vet Res. 2023 Feb 15;54:12. doi: 10.1186/s13567-023-01144-2 (PMC9933378; doi:10.1186/s13567-023-01144-2)
Supplement: Supplementary file 3 — Additional file 3. Primary antibodies used for immunohistochemistry. [file 13567_2023_1144_MOESM3_ESM.docx]

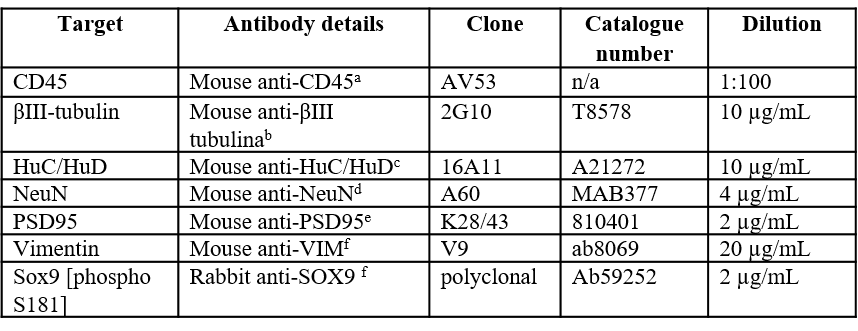


**Additional file 3 Primary antibodies used for immunohistochemistry.**

^a^ Institute for Animal Health; Garceau et al. 2015 [doi.org/10.1186/s12915-015-0121-9](http://doi.org/10.1186/s12915-015-0121-9)

^b^ Sigma-Aldrich; Lee et al*.* 1990 [doi.org/10.1002/cm.970170207](https://doi.org/10.1002/cm.970170207)

^c^ Invitrogen; Ratie et al. 2014 [doi.org/10.3389/fnana.2014.00158](https://doi.org/10.3389/fnana.2014.00158)

^d^ Chemicon; Garcia-Moreno & Molnar 2015 [doi.org/10.1073/pnas.1506377112](https://doi.org/10.1073/pnas.1506377112)

^e^ Biolegend

^f^ Abcam
