# Supplementary material for: Temporal transcriptome profiling of floating apical out chicken enteroids suggest stability and reproducibility
Source: Vet Res. 2023 Feb 15;54:12. doi: 10.1186/s13567-023-01144-2 (PMC9933378; doi:10.1186/s13567-023-01144-2)

Cluster 0001 (1190 nodes)


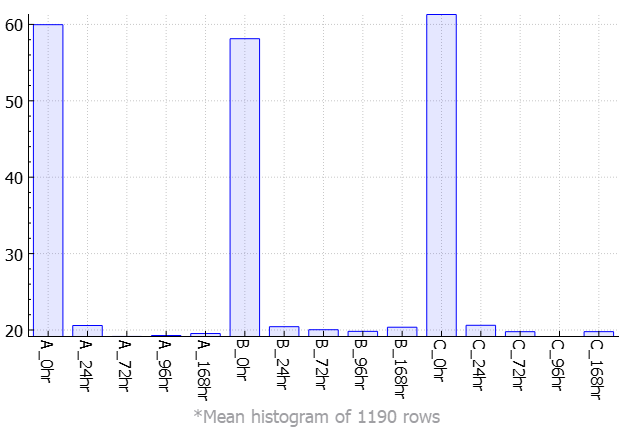


Cluster 0002 (824 nodes)
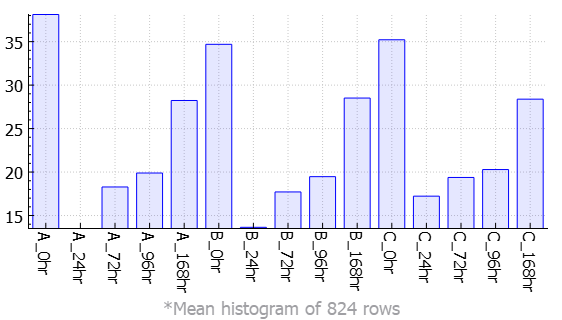


Cluster 0003 (704 nodes)
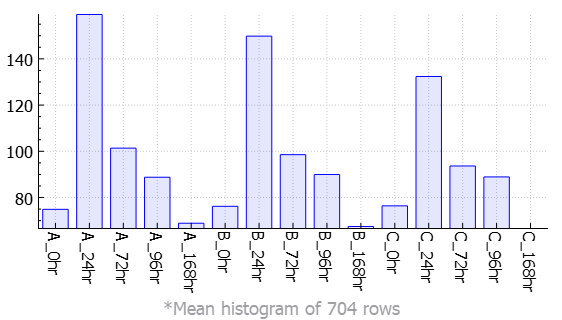


Cluster 0004 (658 nodes)
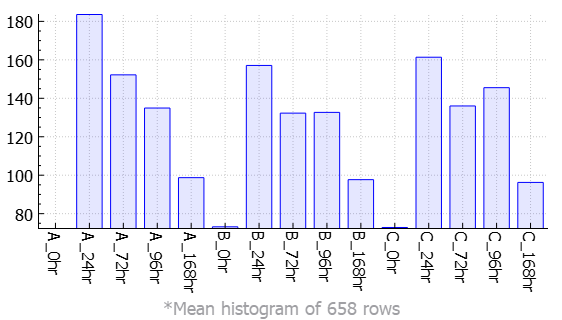


Cluster 0005 (491 nodes)
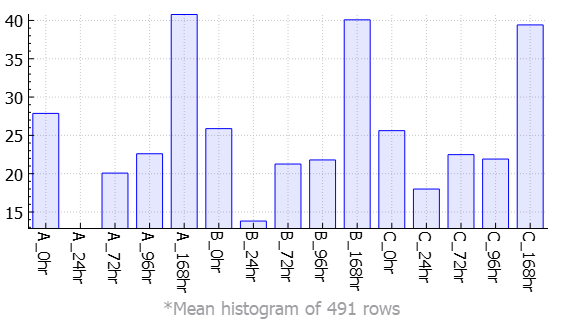


Cluster 0006 (355 nodes)


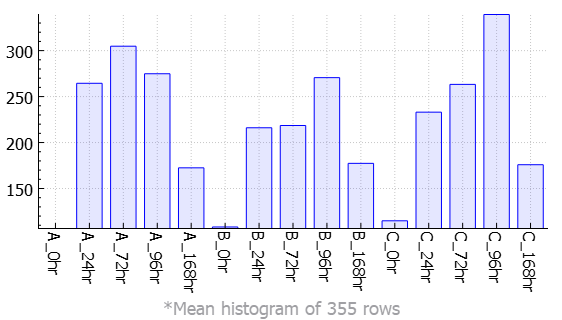


Cluster 0007 (320 nodes)
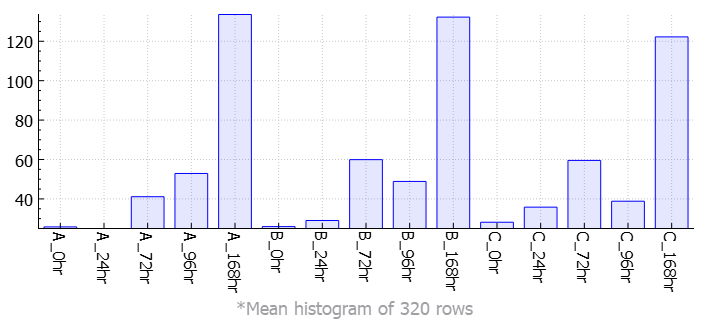


Cluster 0008 (194 nodes)
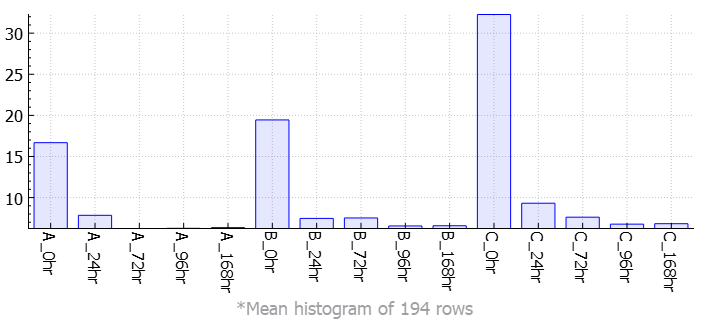


Cluster 0009 (179 nodes)
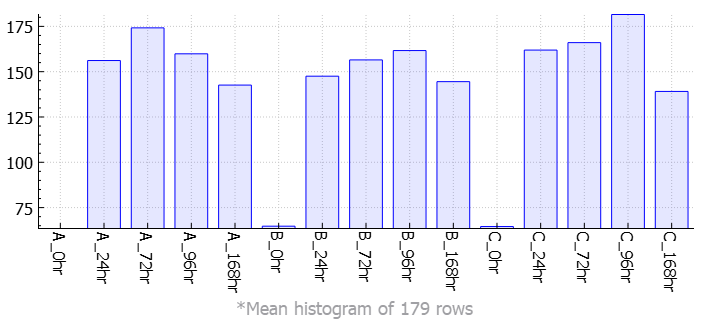


Cluster 0010 (159 nodes)
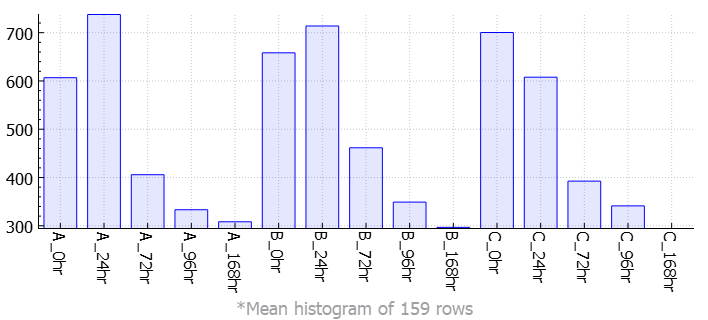


Cluster 0011 (143 nodes)
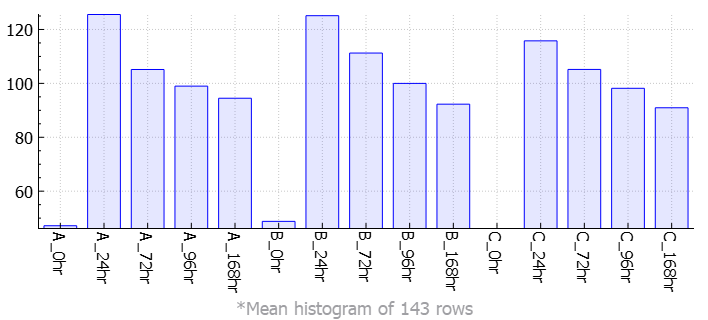


Cluster 0012 (131 nodes)


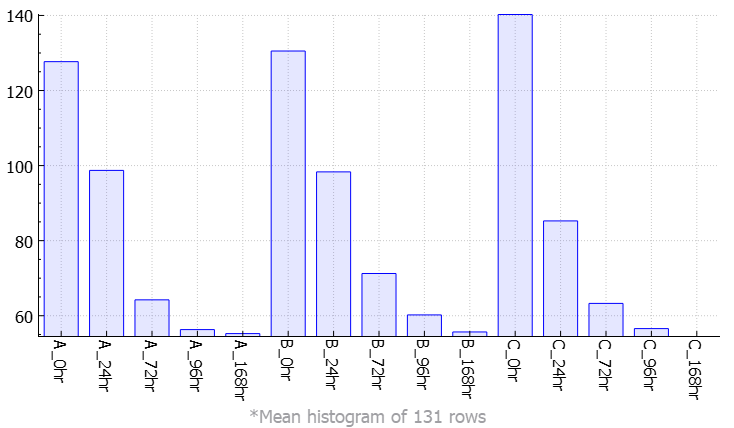


Cluster 0013 (109 nodes)
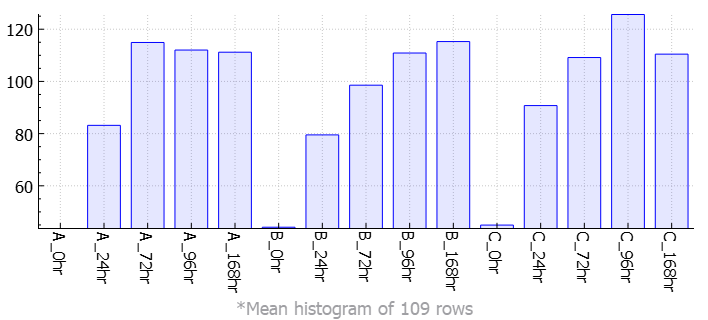


Cluster 0014 (100 nodes)
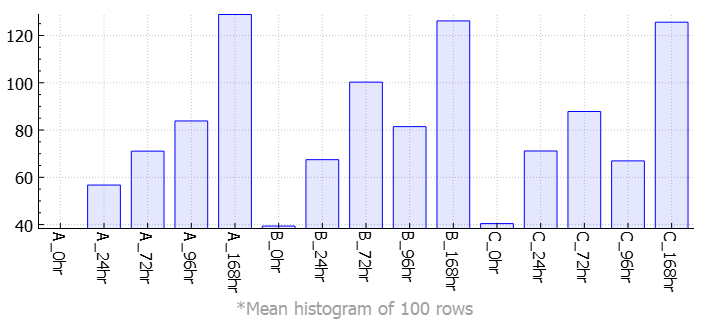


Cluster 0015 (92 nodes)
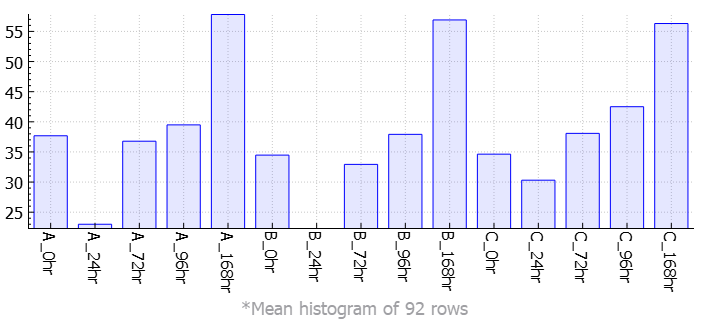


Cluster 0016 (88 nodes)
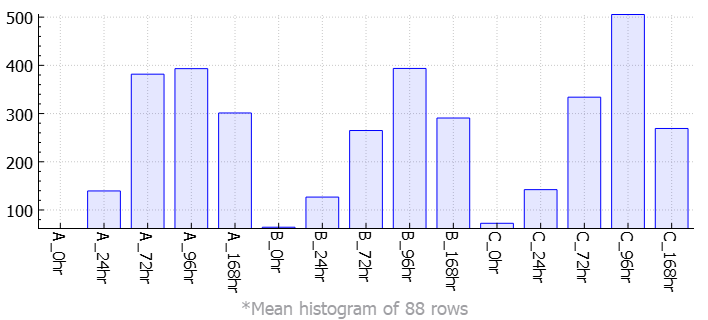


Cluster 0017 (76 nodes)
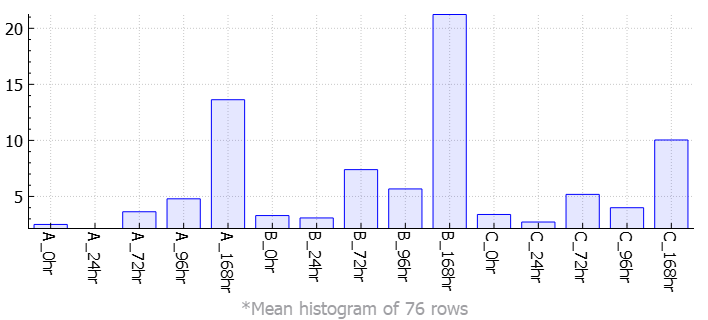


Cluster 0018 (69 nodes)
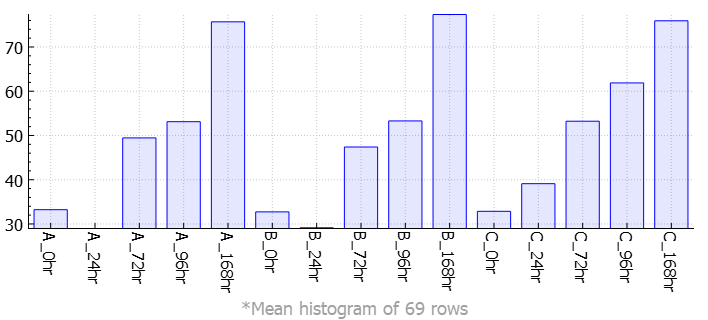


Cluster 0019 (69 nodes)
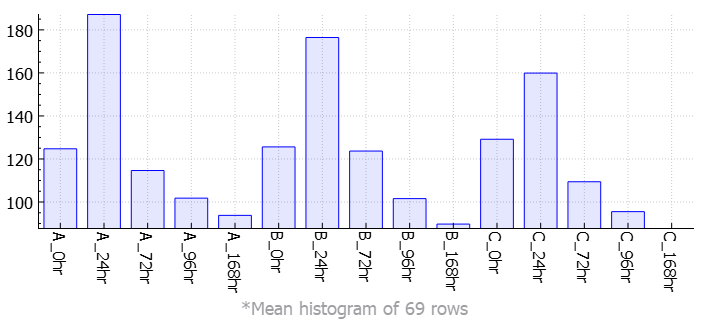


Cluster 0020 (64 nodes)
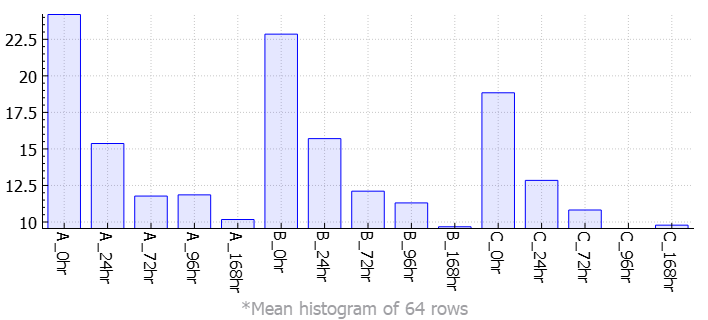


Cluster 0021 (47 nodes)
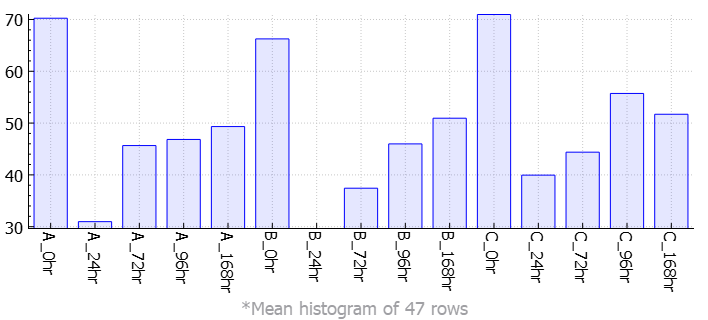


Cluster 0022 (44 nodes)


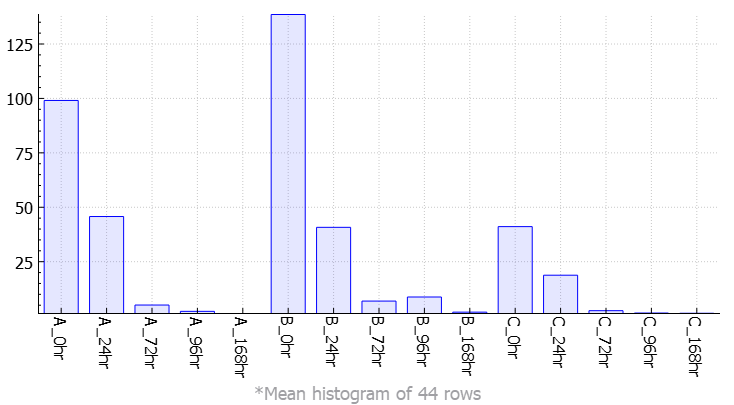


Cluster 0023 (44 nodes)
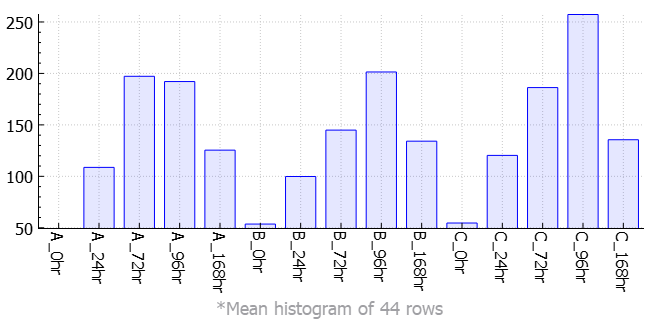


Cluster 0024 (44 nodes)
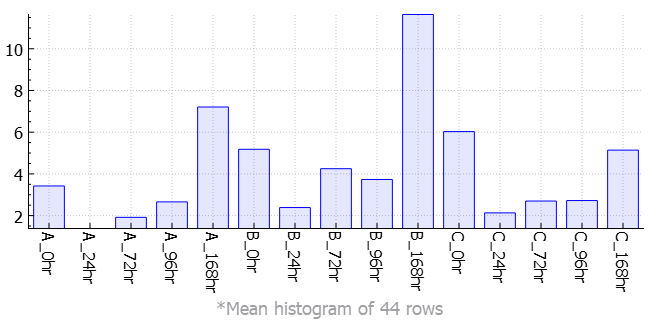


Cluster 0025 (40 nodes)
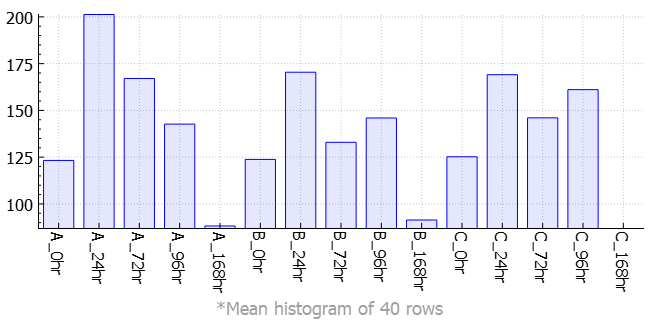


Cluster 0026 (36 nodes)
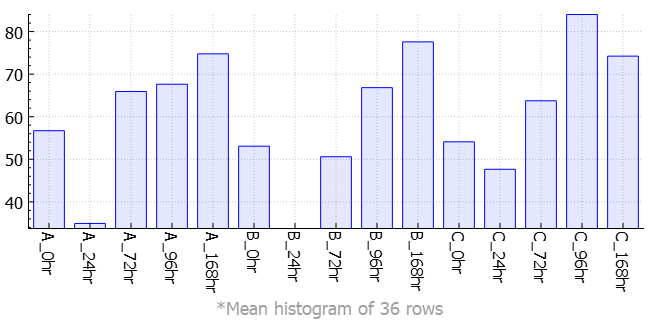


Cluster 0027 (36 nodes)
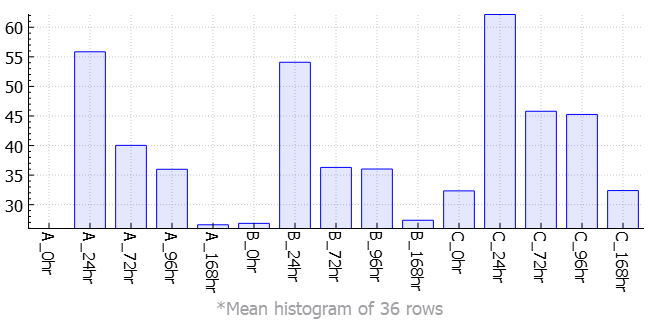


Cluster 0028 (34 nodes)
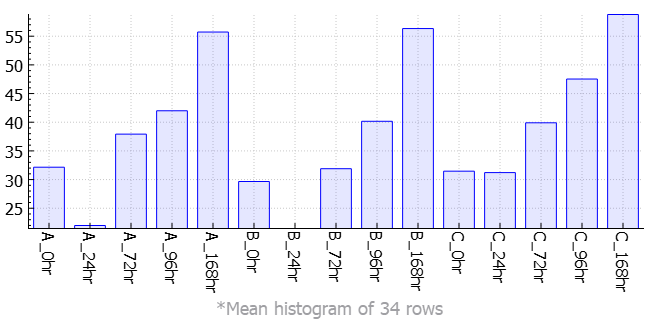


Cluster 0029 (30 nodes)
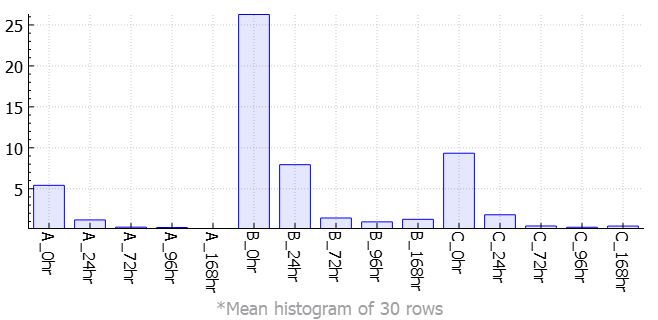


Cluster 0030 (30 nodes)
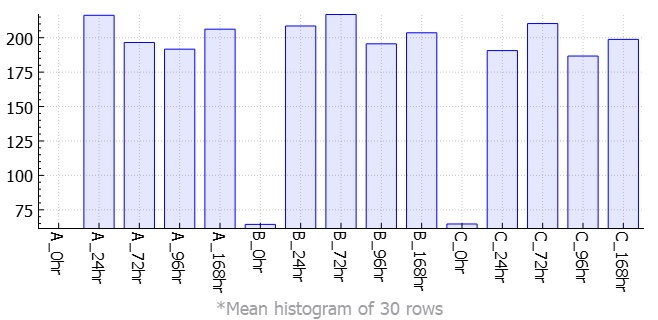


Cluster 0031 (28 nodes)
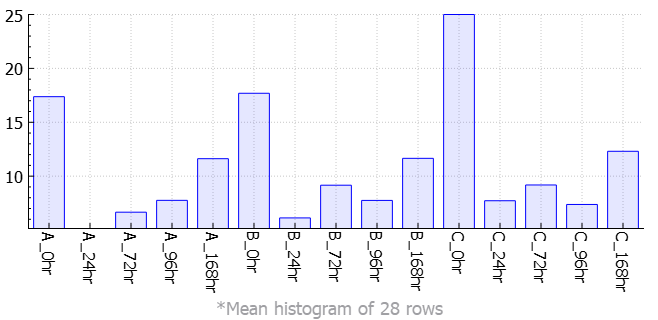


Cluster 0032 (27 nodes)


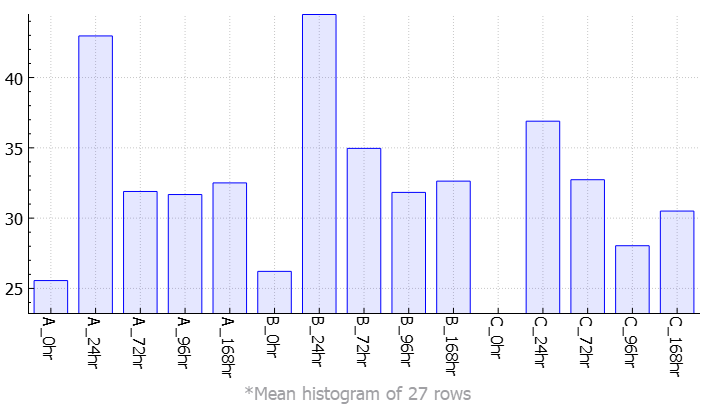


Cluster 0033 (26 nodes)
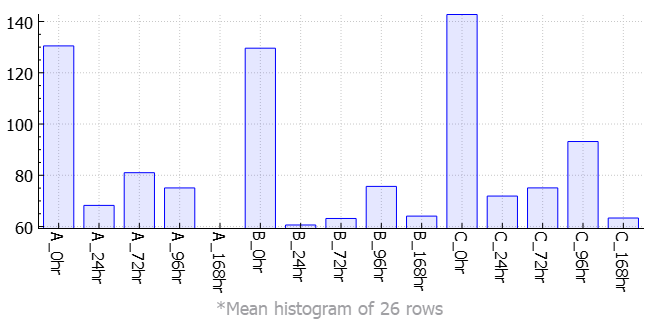


Cluster 0034 (25 nodes)
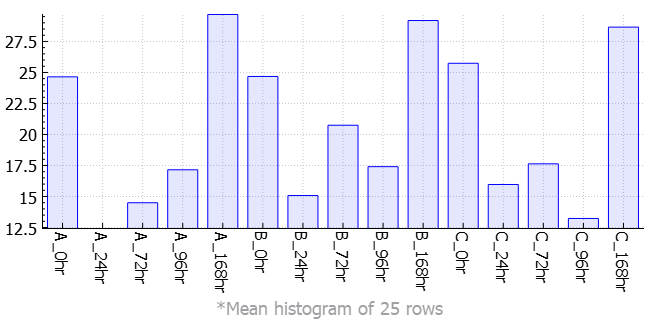


Cluster 0035 (24 nodes)
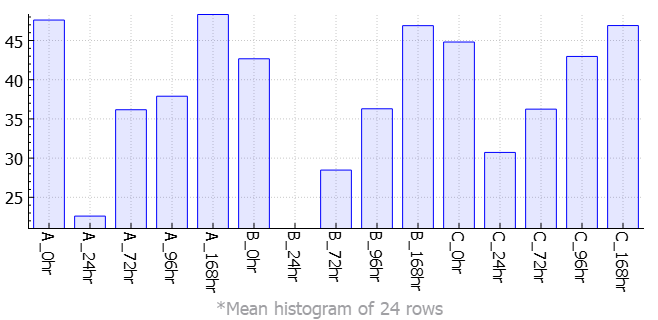


Cluster 0036 (22 nodes)
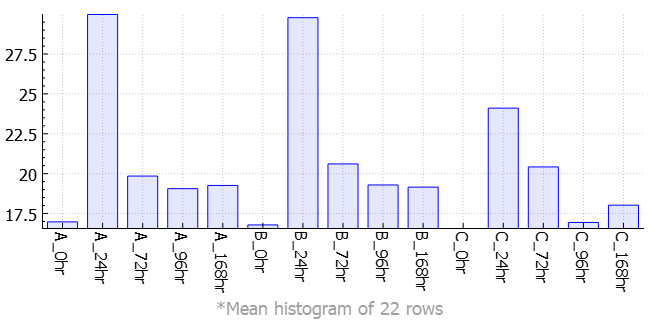


Cluster 0037 (22 nodes)
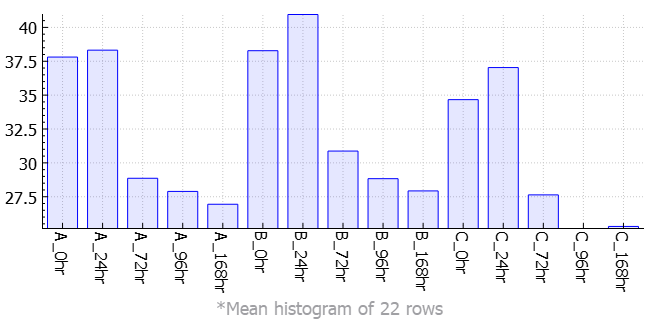


Cluster 0038 (22 nodes)
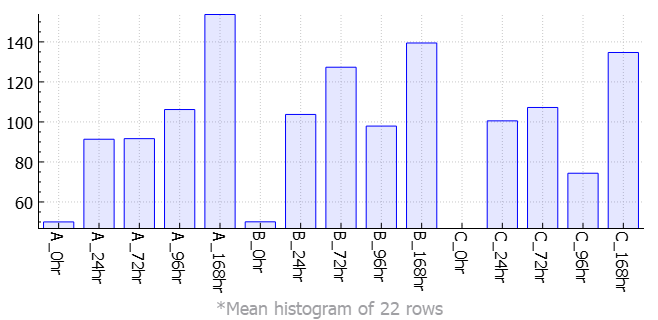


Cluster 0039 (22 nodes)
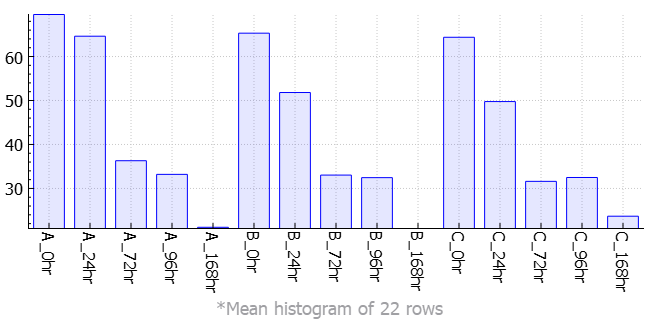


Cluster 0040 (20 nodes)
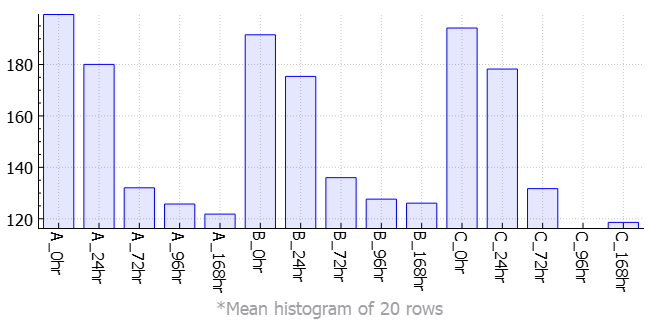


Cluster 0041 (19 nodes)


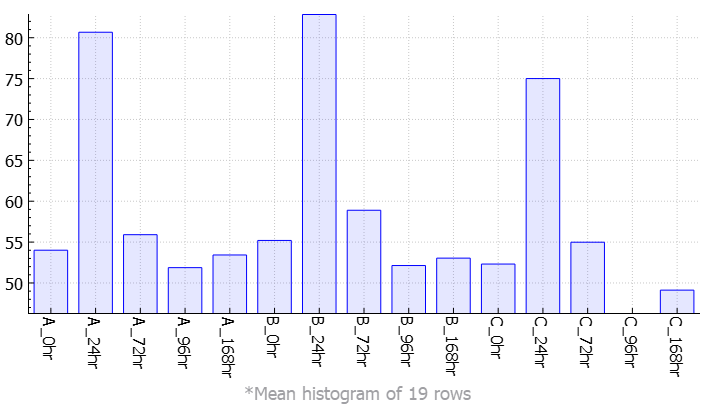


Cluster 0042 (18 nodes)
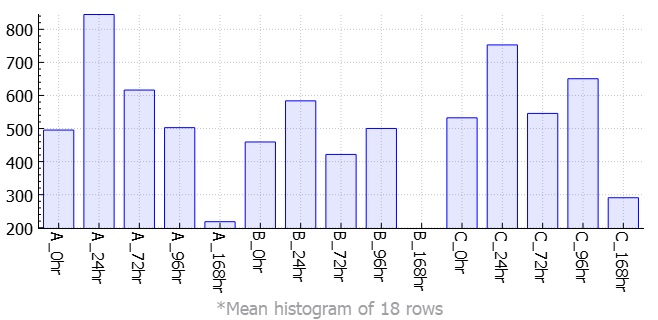


Cluster 0043 (18 nodes)
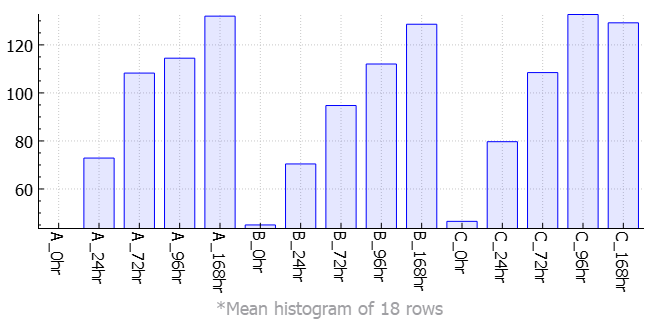


Cluster 0044 (18 nodes)
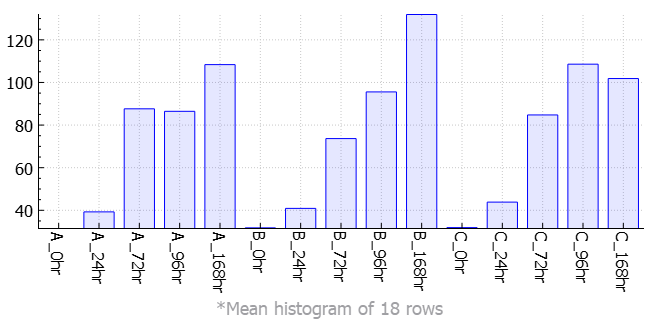


Cluster 0045 (18 nodes)
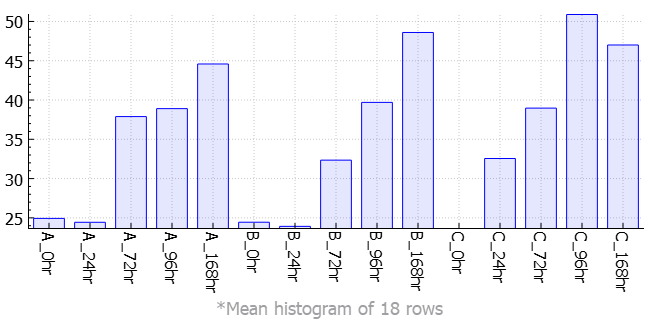


Cluster 0046 (18 nodes)
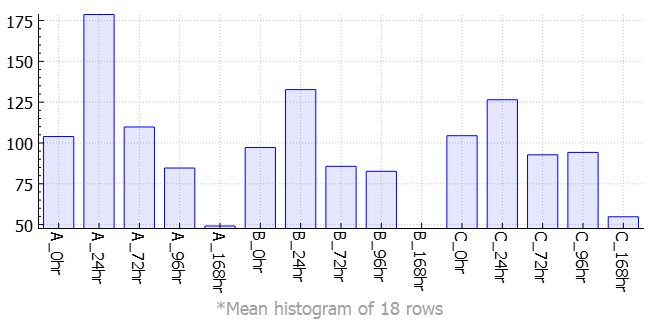


Cluster 0047 (17 nodes)
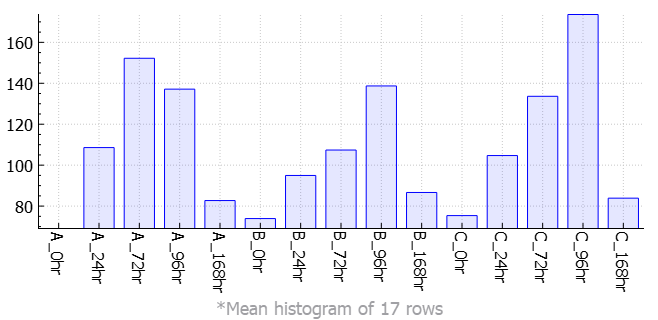


Cluster 0048 (17 nodes)
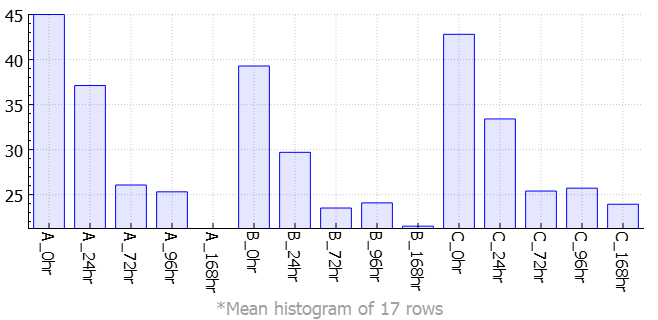


Cluster 0049 (17 nodes)
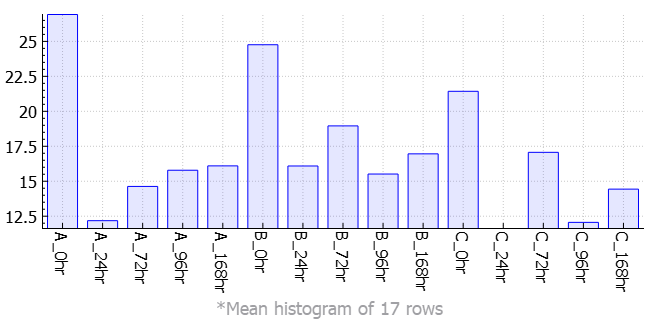


Cluster 0050 (17 nodes)
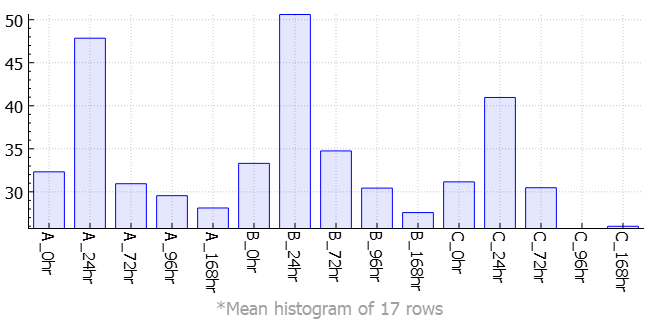

Supplement: Supplementary file 5 — Additional file 5. Mean expression profiles of the genes in each of the largest 50 co-expression clusters. Individual mean expression profiles of the genes in each of the largest 50 co-expression clusters derived from the network graph. The x axis shows the samples ordered by time of cultures. The y axis shows the mean expression intensity (transcripts/million reads, TPM) for the cluster. [file 13567_2023_1144_MOESM5_ESM.docx]
