# Supplementary material for: Temporal transcriptome profiling of floating apical out chicken enteroids suggest stability and reproducibility
Source: Vet Res. 2023 Feb 15;54:12. doi: 10.1186/s13567-023-01144-2 (PMC9933378; doi:10.1186/s13567-023-01144-2)
Supplement: Supplementary file 7 — Additional file 7. Proliferation in the enteroids. Graph demonstrating the percentage of proliferating (EdU+) cells within enteroids over 7 days of culture, alongside z-stack image of proliferating (EdU+) cells within a 14 h enteroid. [file 13567_2023_1144_MOESM7_ESM.pptx]

## Slide 1
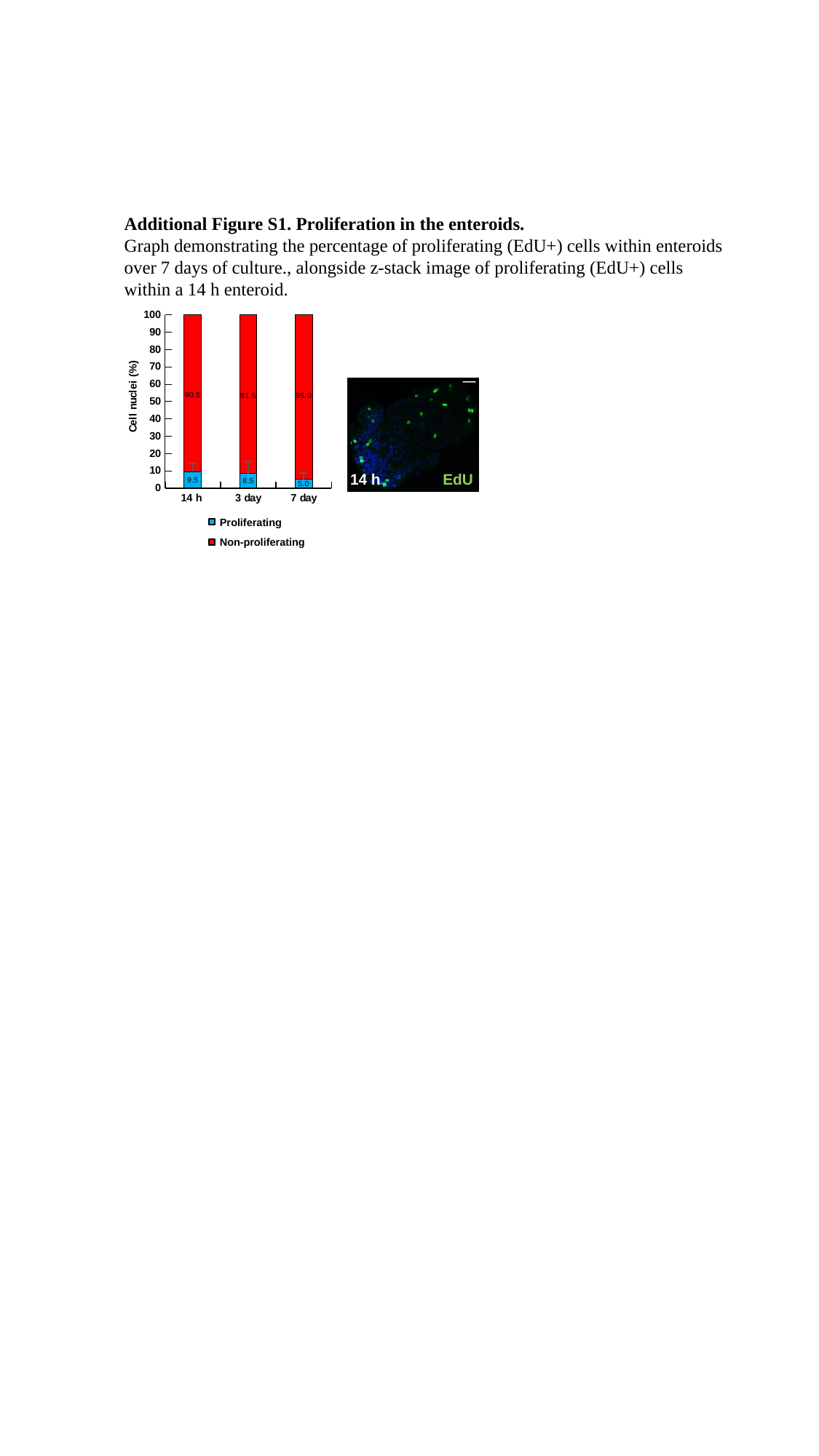

Additional Figure S1. Proliferation in the enteroids.
Graph demonstrating the percentage of proliferating (EdU+) cells within enteroids over 7 days of culture., alongside z-stack image of proliferating (EdU+) cells within a 14 h enteroid.
### Chart
| Category | proliferating | non-proliferating |
|---|---|---|
| 14 h | 9.51 | 90.49 |
| 3 day | 8.52 | 91.48 |
| 7 day | 5.02 | 94.98 |
Proliferating
Non-proliferating
14 h
EdU
