# Supplementary material for: Temporal transcriptome profiling of floating apical out chicken enteroids suggest stability and reproducibility
Source: Vet Res. 2023 Feb 15;54:12. doi: 10.1186/s13567-023-01144-2 (PMC9933378; doi:10.1186/s13567-023-01144-2)
Supplement: Supplementary file 8 — Additional file 8. Cell-type gene expression in chicken enteroid cultures. Expression of stem cell, Paneth cell, enteroendocrine cell, goblet cell and enterocyte genes in 1 day chicken enteroid cultures. Confocal images of chicken enteroids at 2 days of culture stained for. [file 13567_2023_1144_MOESM8_ESM.pptx]

## Slide 1
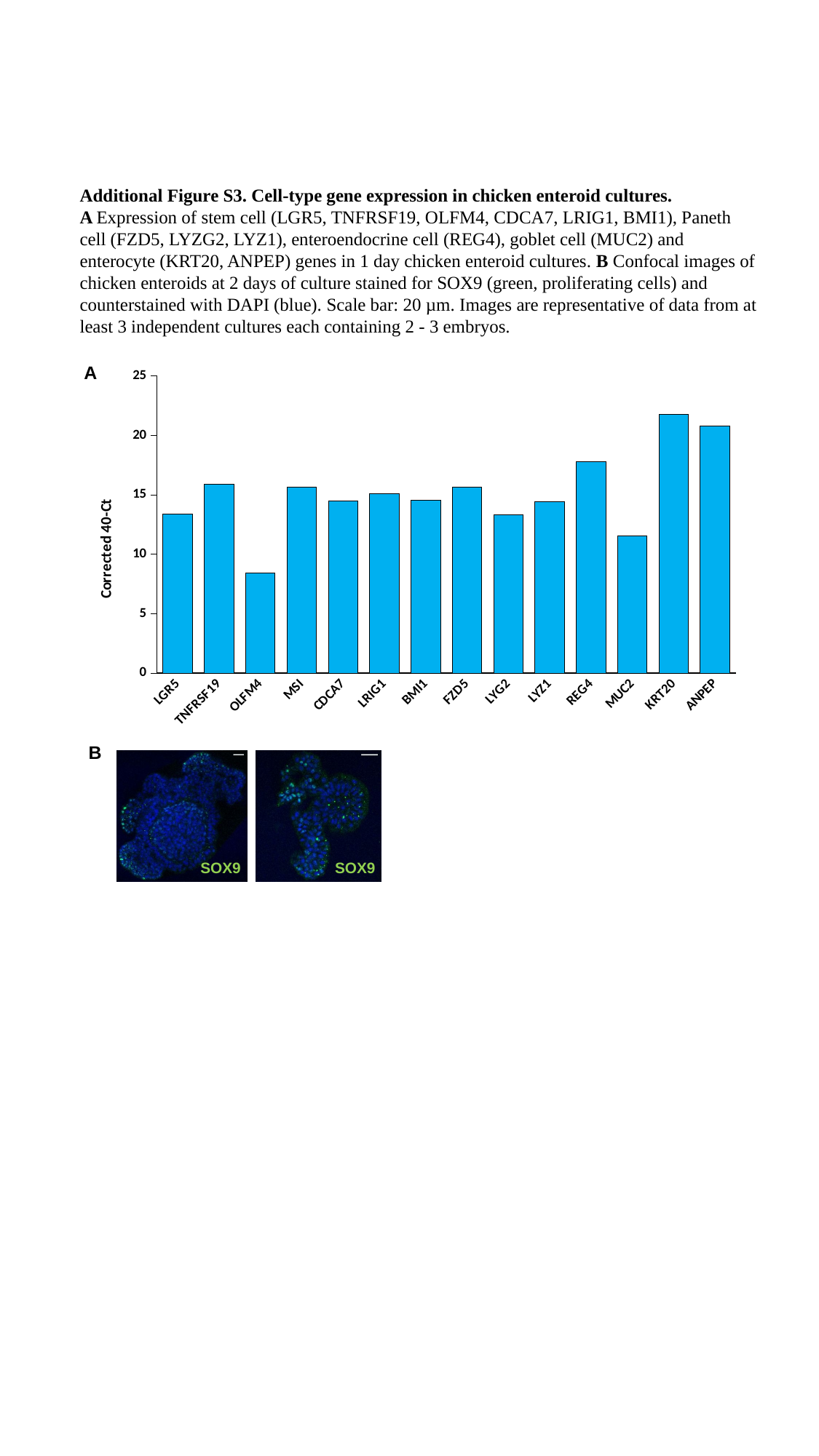

Additional Figure S3. Cell-type gene expression in chicken enteroid cultures.
A Expression of stem cell (LGR5, TNFRSF19, OLFM4, CDCA7, LRIG1, BMI1), Paneth cell (FZD5, LYZG2, LYZ1), enteroendocrine cell (REG4), goblet cell (MUC2) and enterocyte (KRT20, ANPEP) genes in 1 day chicken enteroid cultures. B Confocal images of chicken enteroids at 2 days of culture stained for SOX9 (green, proliferating cells) and counterstained with DAPI (blue). Scale bar: 20 µm. Images are representative of data from at least 3 independent cultures each containing 2 - 3 embryos.
A
### Chart
| Category | 40-ct |
|---|---|
| LGR5 | 13.403400000000001 |
| TNFRSF19 | 15.87671 |
| OLFM4 | 8.432379999999998 |
| MSI | 15.671869999999998 |
| CDCA7 | 14.493929999999999 |
| LRIG1 | 15.12714 |
| BMI1 | 14.530259999999998 |
| FZD5 | 15.665030000000002 |
| LYG2 | 13.35096 |
| LYZ1 | 14.40248 |
| REG4 | 17.78522 |
| MUC2 | 11.551459999999999 |
| KRT20 | 21.75248 |
| ANPEP | 20.80263 |B
SOX9
SOX9
