# Supplementary material for: Temporal transcriptome profiling of floating apical out chicken enteroids suggest stability and reproducibility
Source: Vet Res. 2023 Feb 15;54:12. doi: 10.1186/s13567-023-01144-2 (PMC9933378; doi:10.1186/s13567-023-01144-2)
Supplement: Supplementary file 10 — Additional file 10. Leucocytes identified in the chicken enteroid lamina propria. Expression of immune cell population gene sets analysed by RNA sequencing analysis. Confocal images of chicken enteroids at 2 days of culture stained for CD45. [file 13567_2023_1144_MOESM10_ESM.pptx]

## Slide 1
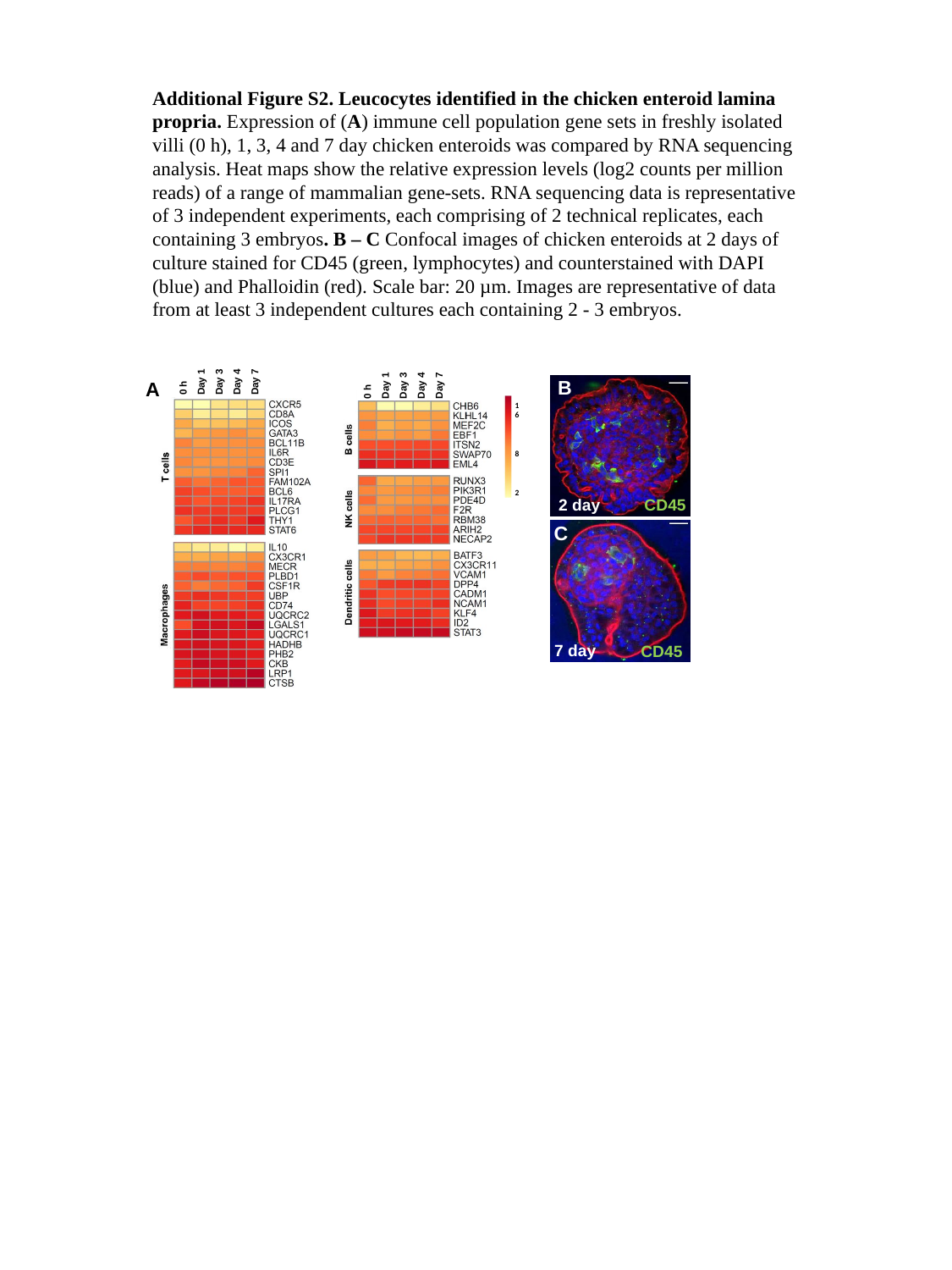

Additional Figure S2. Leucocytes identified in the chicken enteroid lamina propria. Expression of (A) immune cell population gene sets in freshly isolated villi (0 h), 1, 3, 4 and 7 day chicken enteroids was compared by RNA sequencing analysis. Heat maps show the relative expression levels (log2 counts per million reads) of a range of mammalian gene-sets. RNA sequencing data is representative of 3 independent experiments, each comprising of 2 technical replicates, each containing 3 embryos. B – C Confocal images of chicken enteroids at 2 days of culture stained for CD45 (green, lymphocytes) and counterstained with DAPI (blue) and Phalloidin (red). Scale bar: 20 µm. Images are representative of data from at least 3 independent cultures each containing 2 - 3 embryos.
0 h
Day 1
Day 3
Day 4
Day 7
0 h
Day 1
Day 3
Day 4
Day 7
A
16
8
2
B
2 day
CD45
C
7 day
CD45
